# New Perspectives on the Importance of Cell-Free DNA Biology

**DOI:** 10.3390/diagnostics12092147

**Published:** 2022-09-03

**Authors:** Abel J. Bronkhorst, Vida Ungerer, Angela Oberhofer, Sophie Gabriel, Eleni Polatoglou, Hannah Randeu, Carsten Uhlig, Heiko Pfister, Zsuzsanna Mayer, Stefan Holdenrieder

**Affiliations:** Munich Biomarker Research Center, Institute for Laboratory Medicine, German Heart Centre, Technical University Munich, Lazarettstraße 36, D-80636 Munich, Germany

**Keywords:** cell-free DNA, circulating tumor DNA, liquid biopsy, cfDNA, ctDNA

## Abstract

Body fluids are constantly replenished with a population of genetically diverse cell-free DNA (cfDNA) fragments, representing a vast reservoir of information reflecting real-time changes in the host and metagenome. As many body fluids can be collected non-invasively in a one-off and serial fashion, this reservoir can be tapped to develop assays for the diagnosis, prognosis, and monitoring of wide-ranging pathologies, such as solid tumors, fetal genetic abnormalities, rejected organ transplants, infections, and potentially many others. The translation of cfDNA research into useful clinical tests is gaining momentum, with recent progress being driven by rapidly evolving preanalytical and analytical procedures, integrated bioinformatics, and machine learning algorithms. Yet, despite these spectacular advances, cfDNA remains a very challenging analyte due to its immense heterogeneity and fluctuation in vivo. It is increasingly recognized that high-fidelity reconstruction of the information stored in cfDNA, and in turn the development of tests that are fit for clinical roll-out, requires a much deeper understanding of both the physico-chemical features of cfDNA and the biological, physiological, lifestyle, and environmental factors that modulate it. This is a daunting task, but with significant upsides. In this review we showed how expanded knowledge on cfDNA biology and faithful reverse-engineering of cfDNA samples promises to (i) augment the sensitivity and specificity of existing cfDNA assays; (ii) expand the repertoire of disease-specific cfDNA markers, thereby leading to the development of increasingly powerful assays; (iii) reshape personal molecular medicine; and (iv) have an unprecedented impact on genetics research.

## 1. Introduction

**Figure 1:**
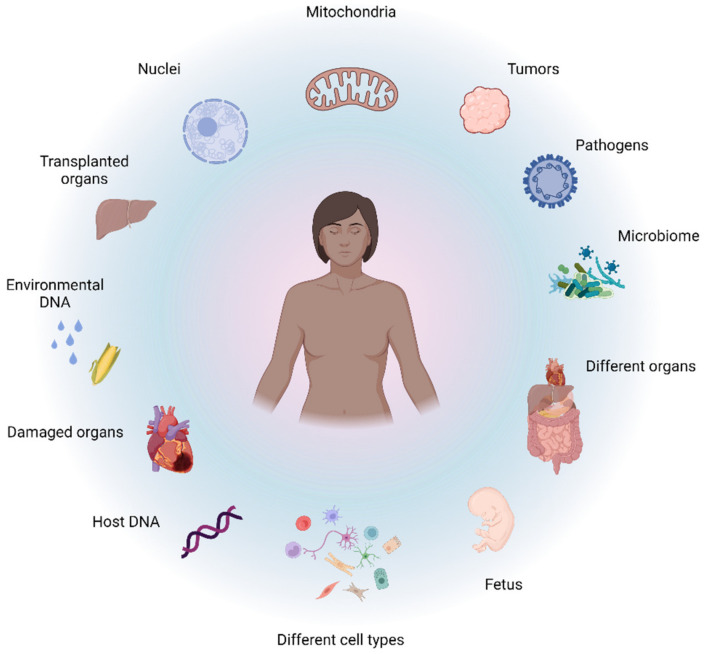
The diverse possible origins of cfDNA in humans. Through various pathways of cell death, clearance, and regulated release, whole or partial genomes of diverse origins are constantly shed into human body fluids in the form of fragmented cfDNA.

Most human body fluids are, through a complex network of release and clearance mechanisms [1], constantly replenished with a population of genetically diverse cell-free DNA (cfDNA) fragments (Figure 1). Since cfDNA samples can be obtained in a one-off and serial fashion through minimally-invasive procedures, e.g., through a blood draw; cfDNA profiling represents an unprecedented treasure trove of real-time genetic data minable for wide-ranging diagnostic, prognostic, and theranostic purposes. Remarkable progress has already been made on this front with the development of cfDNA assays that trump many of the inherent limitations of traditional methods and are slowly transforming the way in which solid tumors, fetal genetic abnormalities, organ transplant rejections, and infections are diagnosed, monitored, and treated. Furthermore, profiling of cfDNA from serial biospecimen collections holds the potential to revolutionize the characterization of temporal genome dynamics in a variety of contexts. The vast dimension of temporal genomic information accessible through cfDNA analysis has, for example, already been tapped towards the development of methods for the longitudinal assessment of various aspects of tumor biology, including residual disease, metastases, intratumor genetic heterogeneity, shifting mutational landscapes, genetic responses to chemo- and radiotherapy, and mechanisms that underlie the emergence of therapy resistance. All of the abovementioned information, which is virtually impossible to procure through tissue biopsies, has been invaluable in guiding therapeutic regimes and has already had an overwhelmingly positive effect on the management and survival of cancer patients (reviewed in [2]). These successes indicate the intriguing possibility of developing serial cfDNA tests for monitoring many other pathologies. First, serial cfDNA analyses may provide new insight into many slowly progressive or chronic illnesses which have been correlated with aberrant cfDNA profiles, such as cardiovascular disease, diabetes, autoimmunity, and neurodegenerative disease. Second, serial cfDNA analyses may be especially useful for monitoring progressive diseases or clinical scenarios that are characterized by rather tight temporal thresholds around rapid malignant transformation or the sudden onset of detrimental effects, such as Parkinson’s disease, Alzheimer’s disease, sepsis, stroke, traumatic injuries, and adverse effects of gene therapy. Moreover, serial analysis of cfDNA may be useful for studying the role of the gut microbiome in human health and disease, the biological footprint and effects of assimilated environmental DNA, and may even have applications in forensic casework and biobank management.

Beyond their use as clinical biomarkers, cfDNA molecules demonstrate underappreciated biological functionality. On one hand, numerous studies have implicated cfDNA in the development, progression, and treatment-resistance of various pathologies, such as cancer, autoimmunity, and COVID-19. Therefore, an improved understanding of the molecular mechanisms that underlie the detrimental effects of cfDNA will grant deeper insight into the pathogenesis of specific diseases and likely reveal currently unknown links between cfDNA and other diseases. At the same time, targeted elimination of cfDNA molecules may represent a new therapeutic modality. On the other hand, empirical evidence and elegant theoretical deliberations indicate that cfDNA may be a hugely underestimated factor in several important biological processes, such as immunity [3], intercellular communication [4], and even evolution [5].

**Figure 2:**
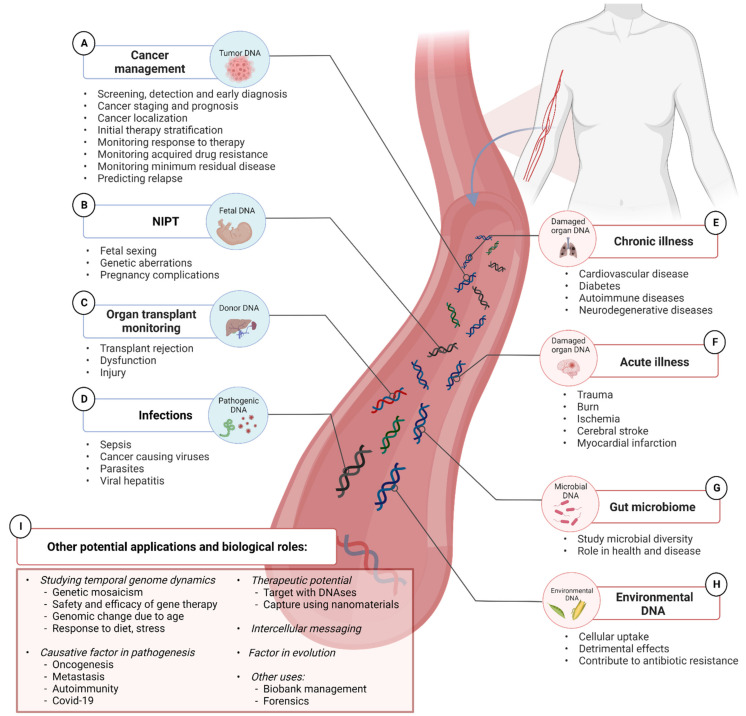
Clinical applications and potential roles of cfDNA in human biology. Human body fluids are constantly replenished by cfDNA fragments of various origins. CfDNA profiling thus offers the unique opportunity to reconstruct major portions of the host- and metagenome, and this information can be harnessed to develop tests for the diagnosis, prognosis, and monitoring of wide-ranging pathologies, such as (**A**) various cancer indications, (**B**) fetal genetic abnormalities and pregnancy complications, (**C**) organ transplant complications, (**D**) infections, (**E**) chronic illnesses, and (**F**) acute illnesses. CfDNA profiling may also be used to characterize (**G**) the gut microbiome and (**H**) assimilated environmental DNA. (**I**) Beyond its use as a clinical biomarker, cfDNA may have many other potential uses and roles in human biology and pathology.

Taken together, cfDNA not only shows significant potential as a surrogate marker for numerous disease indications but is also delicately poised as both a pathological factor and important molecule in several biological processes (Figure 2).

Despite the obvious significance of cfDNA in human biology and pathology, the translation of cfDNA research into useful clinical tests has been advancing at a sub-optimal rate, while the biological functionality of cfDNA is poorly understood and understudied [1,3,6]. As reviewed elsewhere [2,7,8], the development and implementation of clinically meaningful tests is hampered by an array of persistent obstacles that have yet to be overcome, such as a lack of universal preanalytical standards, limited best practice guidelines, analytical limitations, no standard reference materials, insufficient analytical validation, and inadequate clinical trials. Another major factor that negatively impacts both translational and basic cfDNA research relates to the difficulties in achieving high-fidelity reverse engineering of both the quantitative and qualitative characteristics of cfDNA molecules in a biospecimen. Much progress has been made in this regard through rapidly evolving technology, molecular methods, integrated bioinformatics, and machine learning (ML) algorithms, coupled with major efforts in optimizing and standardizing preanalytical procedures. However, accurate measurements of cfDNA are still challenged by numerous obstacles that relate to the biological characteristics of cfDNA. The most prominent challenges in this regard are: (i) the immense heterogeneity in the characteristics of cfDNA in vivo, and the difficulties in differentiating analytically between different cfDNA types (ii) the complex network of biological, physiological, pathological, lifestyle, and environmental factors that modulate the characteristics of cfDNA, (iii) the existence of numerous possible preanalytical steps that are biased toward the preservation, degradation, elimination, or capture of specific cfDNA subtypes, and (iv) a poor understanding of all of the former. Therefore, in this review we explored the biological features of cfDNA and show how a deep and structured enquiry into cfDNA biology may (a) augment the sensitivity and specificity of currently existing cfDNA assays, especially clinical tests based on the detection of hotspot mutations, (b) expand the repertoire of disease-specific cfDNA markers, thereby leading to the development of new and more powerful assays and thus significantly expand the liquid biopsy toolbox and clinical scope of cfDNA assays, (c) open an unprecedented window of access for studying temporal genomic changes as it relates to a wide range of processes, and (d) ultimately shed new light on poorly understood processes as well as reveal hidden biological processes, likely catalyzing a surge of new discoveries about genome function.

## 2. Measurement of Total cfDNA Levels in Different Contexts

### 2.1. Serious Medical Conditions

Elevated total cfDNA has been detected in a wide range of disorders, such as cancer [2,9], autoimmune diseases [10] (e.g., systemic lupus erythematosus [11,12], rheumatoid arthritis [13], and systemic sclerosis [14]), trauma patients [15] (e.g., brain injuries [16,17] and burn patients [18]), cardiovascular diseases (e.g., acute myocardial infarction [19] and acute coronary syndrome (ACS) [20]), viral infections (e.g., acute Puumala Hantavirus Infection [21] and Crimean–Congo hemorrhagic fever (CCHF) [22]), benign gastrointestinal tract disorders [23,24,25], kidney disease [26,27], lung disease (e.g., chronic obstructive pulmonary disease exacerbations [28] and pulmonary embolism [29]), thyroid disease [30], pregnancy disorders (e.g., abnormal placentation, such as preeclampsia [31] and intrahepatic cholestasis [32]), skin conditions (e.g., psoriasis [33,34]), and stroke [35,36].

### 2.2. Other Clinical Scenarios

In addition to the more common medical conditions listed above, which are clearly associated with cell or tissue injury and damage, total cfDNA levels have also been correlated with a variety of other more obscure clinical scenarios. Elevated total cfDNA levels have been found in psychiatric disorders, e.g., schizophrenia [37]. Conversely, cfDNA levels were found to be lower in patients with extra temporal lobe epilepsy vs. healthy subjects [38]. CfDNA levels were also found to be correlated with indications during in vitro fertilization (IVF) procedures, while also reflecting male and female fertility status. For example, cfDNA levels in the fluid of the blastocoel cavity of embryos correlated positively with embryo morphology, indicating promise as a candidate marker of embryo quality and implantation potential [39]. Similarly, increased cfDNA levels have been demonstrated in low pregnancy rates among women undergoing IVF–embryo transfer [40]. In line with this, the performance of various stress reduction techniques among women undergoing infertility treatment resulted in a decrease in total cfDNA levels [41]. Interestingly, cfDNA levels in the seminal plasma of azoospermic (no sperm in ejaculate) men has been shown to be higher than in normozoospermic men [42,43].

### 2.3. Medical Treatments and Therapy

Various kinds of medical treatments have been shown to result in elevated cfDNA levels in comparison with untreated subjects, including various surgeries [44], radiotherapy [44], high doses of corticosteroids, particularly in patients with lymphoid hyperplasia or sustained immunologic stress (which may be ascribed to the lympholytic effect of steroids) [45], patients with end-stage renal disease (ESRD) receiving haemodialysis [27,46,47], and intensive treatment unit patients receiving ventilation [48,49].

### 2.4. Different Physiological States

Studies have demonstrated higher cfDNA levels in older individuals (over 60 years) vs. younger individuals, a phenomenon linked with various age-associated processes, including increased cellular senescence, inflammation-induced cell death, and reduced clearance and phagocytosis capacity [50,51]. Increased mass of adipose tissue in overweight and obese pregnant women has been shown to result in increased cfDNA levels, often complicating non-invasive prenatal screening tests (NIPTs) [52,53].

Some reports suggest that measured cfDNA levels depend on the time of day when samples are collected and show significant intra- and interindividual variation [54,55]. One study, for example, reported that the majority of subjects presented with maximum cfDNA levels at midday [56], while fasting subjects in a different study showed the highest cfDNA levels in the morning, which decreased up to three-fold after breakfast and lunch [57,58]. While it is speculated that circadian rhythms, postprandial effects, or the effects of fluctuating blood lipid content (in response to food intake) on DNA isolation methods may play a role in intra-day fluctuating cfDNA levels, the true cause is not yet clear.

An evaluation of 25 studies that reported on the relationship between cfDNA and biological sex revealed a greater likelihood of increased cfDNA levels in males vs. females. However, these differences are considered to be minor and may reflect lifestyle differences between males and females, and as such requires further investigation [59]. Other variables such as haematocrit or cannula placement pain [60], height [61,62], and the menstrual cycle [63,64] were not found to affect total cfDNA levels.

### 2.5. Lifestyle Factors and Occupational Exposure Hazards

Studies have shown that high-intensity or acute exercise markedly increases plasma cfDNA levels. While the cause of this phenomenon is not yet known, it is possible that these cfDNA molecules originate from strenuous physical exertion-induced muscle damage, oxidative stress-induced DNA damage, or leukocyte inflammatory responses [54,62,65,66,67], but this explanation is challenged by observations that cfDNA levels increase dramatically almost immediately after beginning exercise [54,68]. Conversely, cfDNA measurements in subjects engaging in chronic exercise showed less pronounced spikes and inconsistent levels [69]. To-date, only two studies have investigated the effects of alcohol intake on total cfDNA levels, but no correlation was found [70,71]. However, more research is needed, as these studies had incomplete methodological reporting, used a non-specialized method for cfDNA isolation, failed to specify alcohol intake for subjects in the ‘moderate-severe’ category [71], have not in their cohort categorization accounted for possible differences between social drinkers and non-drinkers [70], and were performed in the context of a study on cancer [59].

Despite the DNA-damaging effects of cigarette smoking, surprisingly few studies have assessed the correlation between smoking and cfDNA levels. Of the ten studies that have been conducted so far, highly conflicting results were obtained, together showing virtually no correlation between smoking and cfDNA levels [59]. It is possible that a correlation may be found in clinical studies that have enrolled appropriately powered, equally balanced, and sex- and age-matched subjects into their cohorts, for example.

Significantly elevated total cfDNA levels have been demonstrated in greenhouse workers exposed to pesticides for 5–15 years vs. controls [72], while increased cfDNA levels coupled with increased DNA damage were demonstrated in workers exposed to toxic paints in automobile paint shops [73]. Conversely, another study demonstrated a much lower level of cfDNA in nuclear-site workers exposed either to gamma-neutron or chronic tritium β-radiation [74]. To our knowledge, no other studies have evaluated the effect of occupation-related exposure to toxic substances on total cfDNA levels. Yet, it seems likely that cfDNA measurements could potentially be used to monitor worker health in various occupations that are at increased risk of chronic exposure to harmful substances. These include, for example, miners that inhale silica, construction workers that are exposed to asbestos and regularly use products that release toxic fumes (e.g., insulation, glues, paint, polyurethane, blown foam, and solvents), welders that work with metals that become toxic when heated, and textile workers exposed to chemicals such as formaldehyde, arsenic, and cadmium.

As mentioned earlier, total cfDNA levels are influenced by some therapies, and may be affected by food intake. Apart from these studies, and those on alcohol use, cigarette smoking, and one study that found no correlation between cfDNA levels and a history of betel nut chewing (seeds with stimulatory effects akin to amphetamines and cocaine) [70], there are no further data on the correlation between nutritional, medicinal, or drug abuse status and changes in total cfDNA levels. Detecting changes in the baseline values of cfDNA in response to the consumption of certain foods, dietary supplements, medicine, or drugs of abuse may be complicated by the short half-life of cfDNA, which is currently estimated between 16 min and 2.5 h [75,76,77]. However, it may, for example, be interesting to investigate changes in total cfDNA levels during the chronic use of medications that may cause muscle atrophy, such as cholesterol medication, or in individuals with a long-term diet of inflammatory foods, or in individuals that abuse drugs that cause chronic inflammation, for example inflammation of the immune system [78] or pancreas [79]. Other lifestyle factors such as frequent blood donation were not found to correlate with cfDNA levels [80].

### 2.6. Limitations of Quantitative cfDNA Measurements

**Figure 3:**
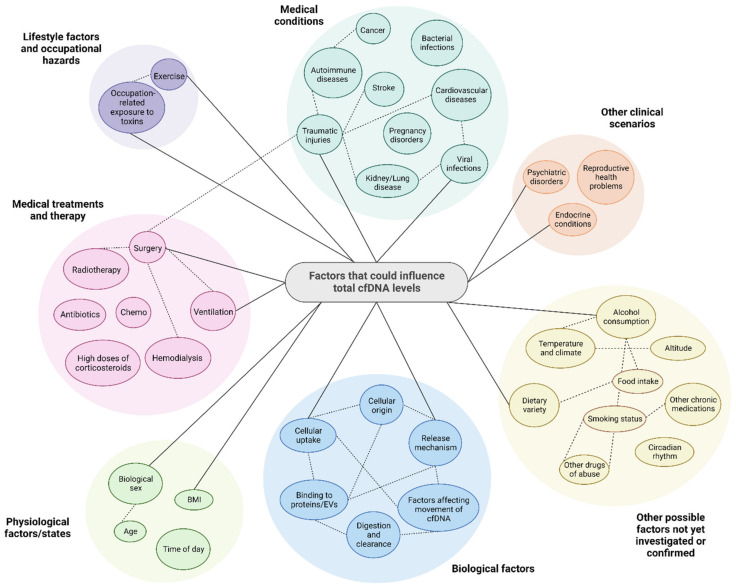
Factors that can potentially affect total cfDNA levels. Here we summarize the wide-ranging factors that have been experimentally shown to modulate total cfDNA levels. We also show several other factors that possibly affect total cfDNA levels, but which have not yet been conclusively demonstrated or have not yet been investigated. This makes it very difficult to correlate total cfDNA levels with a specific factor and limits the clinical use of total cfDNA measurements.

At first glance, the reports discussed in the sections above suggest that total cfDNA levels could potentially serve as a stand-alone biomarker for detecting and monitoring several disorders and other clinical scenarios. However, the use of total cfDNA levels for this purpose is highly unlikely due to the convergence of several factors that result in greatly overlapping data between different disease types and healthy individuals both in individual studies and in interstudy comparisons, thereby precluding the establishment of cut-off values or normal reference ranges for any specific condition (Figure 3). These factors include: (i) elevated cfDNA levels is not a phenomenon unique to specific pathological states but is instead a common consequence of many diseases; (ii) cfDNA levels increase in response to a wide range of ordinary non-pathological conditions and also correlates with numerous lifestyle factors; (iii) as will be described in greater detail in Section 9, accurate measurements of total cfDNA are also significantly affected by a plethora of biological factors as well as factors relating to its physico-chemical properties, the nature of various preanalytical steps, and analytical decisions. It is also likely that the co-presence of so many overlapping variables has resulted in the reporting of erroneous correlations between total cfDNA levels and a specific factor, while interesting correlations between a specific factor and total cfDNA levels have been obscured to date.

Although total cfDNA levels have limited clinical utility, the surge of early studies on the correlation between total cfDNA levels and clinicopathological data sparked widespread interest in the genetic and epigenetic characterization of cfDNA. This primed the ground for rapid advancements in molecular methods and technologies which accelerated the discovery of unknown correlations between qualitative characteristics of cfDNA and various disease indications, particularly in the medical fields of oncology, prenatal testing, and organ transplant monitoring. This, in turn, enabled the development of several cfDNA tests that have been approved for use in routine clinical practice. In the next sections we discuss new advances, approved and potential clinical applications, as well as challenges related to qualitative cfDNA assays. In outlining these new advances in the research field, it is interesting to note how it may reignite interest in the conditions discussed in Section 2.1, Section 2.2, Section 2.3, Section 2.4 and Section 2.5 or inspire enquiry into unexplored domains of biology and medicine in relation to cfDNA, and how quantitative measurements of total cfDNA may be resurrected as a potential auxiliary marker to qualitative characterization of cfDNA.

## 3. Sequence Analysis of cfDNA

Hundreds of studies have investigated cfDNA in various biospecimen types for the detection of mutations or sequences unique to individuals, organisms, and diseases. This led to many exciting discoveries, while the use of increasingly sophisticated molecular analysis methods for cfDNA analysis has enabled the development of diagnostic assays in various clinical fields, some of which are already clinically available. Below we give an overview of the potential applications of cfDNA sequence analysis.

### 3.1. Cancer

The tremendous value of cfDNA as a surrogate molecular marker throughout cancer management is now well understood (reviewed in refs. [2,81,82,83]). In-depth profiling of cancer-specific mutations in cfDNA may enable the development of pan-cancer screening tests for at-risk groups or unsuspected healthy populations, thereby allowing early detection and prompt treatment (reviewed in [8,84,85]). As the level of cancer-specific mutations shows a high correlation with tumor size, quantitative measurements could be used as an indicator of disease stage and may even predict the clinical outcome of patients. As will be discussed in a later section, various cell and tissue-specific information is encoded into both classical and newly discovered epigenetic features of cfDNA, which may not only act as auxiliary markers for tumor detection but may enable clinicians to pinpoint the location of tumors, especially those of unknown primary. Furthermore, cfDNA can be used to guide the selection of new targeted therapies which only work when specific mutations are present. Tissue biopsies collect only a fraction of tumor tissue, and therefore do not capture the spatial genetic heterogeneity of the complete tumor. On the other hand, tumor-derived cfDNA, which is released into body fluids from many parts of the tumor, provides a much more holistic representation of the genetic landscape of the whole tumor. Thus, given the minimally-invasive nature of venipuncture, longitudinal cfDNA sampling enables the assessment of dynamic changes in cfDNA levels, the identification of acquired resistance-conferring mutations, and the tracking of clonal evolution. These temporal data can be harnessed not only to monitor the response of cancer to surgical removal, which makes possible the detection and prediction of minimal residual disease or recurrence, but also monitor the response to other therapies, which makes it possible to detect and study the emergence of acquired resistance. The information provided by cfDNA analysis will facilitate the development of better therapies and inform the selection of more effective therapy regimes.

### 3.2. Fetal Genetic Abnormalities

Since the discovery of the presence of cell-free fetal DNA in maternal plasma, non-invasive prenatal tests have been developed for fetal sexing [86], screening of various fetal genetic abnormalities [87,88], and monitoring pregnancy complications [31], many of which are now routinely screened for (reviewed in ref. [89]).

### 3.3. Organ Transplant Monitoring

Characterization of unique single-nucleotide polymorphisms in cfDNA has allowed the differentiation between host- and recipient-derived cfDNA in organ transplant patients and is emerging as a potentially useful clinical tool for monitoring post-transplant organ rejection, dysfunction, and injury [90,91,92,93,94,95,96].

### 3.4. Detecting Pathogenic DNA

Pathogenic DNA or RNA from bacteria (e.g., *Mycobacterium tuberculosis*-derived DNA [97] and Pneumonia pathogens [98]), viruses (e.g., Crimean–Congo hemorrhagic fever (CCHF) [22]), and parasites (e.g., *Leishmania*, *Plasmodium*, *Schistosoma*, *Trypanosoma,* and *Wuchereria* spp.) have been detected in body fluids [99]. Characterization of pathogenic DNA in body fluids may facilitate the early detection of a wide range of infections. For example, a 24-marker quantitative real-time PCR (qPCR) assay has recently been developed for the detection of sepsis well before the onset of clinical symptoms [100]. Similarly, detection of parasitic cfDNA using nucleic acid amplification tests (NAATs) showed improved accuracy over traditional microscopic and serological diagnostic tests, while the possibility of convenient, cost-effective, non-invasive, and painless collection of parasitic DNA from specimens such as urine and saliva is highly practical for implementation in large-scale epidemiological screening programmes [99]. Furthermore, routine screening of cancer-causing viruses such as the Epstein-Barr virus (EBV), which is associated with nasopharyngeal carcinoma [101,102], or Human papillomavirus (HPV), which is associated with oropharyngeal squamous cell carcinoma [103,104] and cervical cancer [105] may enable early detection and prompt treatment.

### 3.5. Studying the Gut Microbiome

The major role that the gut microbiome plays in human health and disease is becoming increasingly appreciated. The detection of high levels of cfDNA fragments in human plasma that originates from both known and unknown resident microorganisms [106,107,108] suggest that the metagenome encoded into the total cfDNA population may not only be harnessed to study and gain further insights into the gut microbiome, such as the mechanisms by which it contributes to human health or disease or the effects of antibiotics, but also gain deeper insights into the microbial diversity within humans, which appear to be much more diverse that initially thought [108].

### 3.6. Studying Environmental DNA in Humans

Environmental nucleic acids ingested through fluids and meals, such as bacterial or plant DNA, has been found in human body fluids. The fate of these nucleic acids is uncertain, but it is suggested that they may remain in body fluids for extended periods before it is degraded, excreted, or assimilated by cells [109,110]. While the relative contribution of environmental DNA to the total cfDNA population has not yet been determined, it is generally considered to be low. However, foreign cfDNA molecules may have underestimated detrimental effects, as studies have shown that they are able to enter the host cell nuclei and incorporate into the genome [111,112,113,114]. For example, a recent study suggested that cfDNA may facilitate the horizontal transfer of antibiotic resistance genes [110]. While no research has been carried out on the topic, we could speculate here that cfDNA may serve as a potential surrogate marker to study the presence and potential effects of genetically modified crops or animals on human health.

### 3.7. Other Potential Uses

While thorough investigation is lacking, some reports have indicated that cfDNA may have potential use in (i) biobanking; analysis of cfDNA in cord blood plasma has been useful for sample identification [115], (ii) forensic casework; cfDNA has been genetically profiled in samples recovered from externally found body fluids, such as blood, sweat, and feces [116,117,118,119], and (iii) quality control assurance of blood donations [120] or transfusions [121].

## 4. Limitations of cfDNA Hotspot Mutation Analysis

An ever-increasing range of disease-specific signatures can be detected through the characterization of cfDNA sequence information. This constitutes a major breakthrough in the use of molecular tests towards minimally invasive personal medicine. The clinical significance of cfDNA sequence analysis is underscored by several recent breakthrough advancements in the field (Table 1).

Despite these exciting achievements and despite the spectacular progress made in the cfDNA research field in the last two decades, the development and implementation of ctDNA assays into routine diagnostic settings has been slow and challenging. The main reason for this is that the timeline for the development of ctDNA assays that bear the required diagnostic sensitivity and specificity for testing in large-scale clinical trials, and thus the development of kits that are fit for clinical roll-out, is extended by numerous, often non-mutually exclusive factors that challenge the robust analytical detection of ctDNA. The most challenging factor is low absolute amounts of ctDNA molecules in the blood, especially in early disease when small tumors shed miniscule amounts of DNA into extracellular space. While the analytical sensitivity of ctDNA assays have surged tremendously in recent years, the detection of scarce ctDNA molecules in a biospecimen is still challenged by the vast background of non-target molecules originating from diverse origins. The analytical challenges involved in detecting low amounts of ctDNA is further exacerbated by the selection of counter-productive preanalytical steps, such as (a) blood-drawing techniques, processing procedures and storage conditions that result in the degradation of target molecules or dilution by the release of germline DNA, (b) extraction procedures that fail to capture a large portion of ctDNA molecules, either due to general recovery inefficiency (e.g., automated methods extract much less DNA than some manual spin-column methods) or size bias (e.g., ctDNA mutations occur in both long and short fragments, but most kits are biased toward the extraction of short fragments).

Moreover, in the rapidly advancing cfDNA research field, which is currently experiencing an influx of new ideas, discoveries, potential applications, and companies, there is not only an ever-expanding menu of products for each preanalytical step, but also a growing repertoire of analytical techniques and technologies open to selection. Although many of these products and technologies have different degrees of efficiency and bias towards specific sample processing procedures, applications, and different cfDNA subpopulations, they are somewhat arbitrarily used by both basic and translational researchers. This significantly complicates the harmonization of cfDNA preanalytics and analytics among researchers, institutions, and clinics. These issues are increasingly addressed [7,58,122,123,124,125,126,127,128,129], yet preanalytical optimization and standardization remains a major, evolving issue that requires ongoing surveillance and active problem solving. As reviewed elsewhere [130], ctDNA profiling is also challenged by clonal hematopoiesis (CH)-derived cfDNA that bear cancer-specific mutations. This is especially problematic as this is a common phenomenon in both cancer patients [131] and healthy subjects [132,133,134,135]. It is currently not clear how the misdiagnosis of CH-derived mutations in cfDNA as malignancy may be prevented, but some suggest that ctDNA and CH-derived cfDNA may be differentiated on the basis of fragment size, as tumor-derived cfDNA is generally shorter than DNA from other origins [136]. However, as discussed in Section 9, it is also noteworthy that there is a wide range of cfDNA size populations in human body fluids, many of which share overlapping origins.

## 5. Beyond cfDNA Hotspot Mutation Analysis

**Figure 4:**
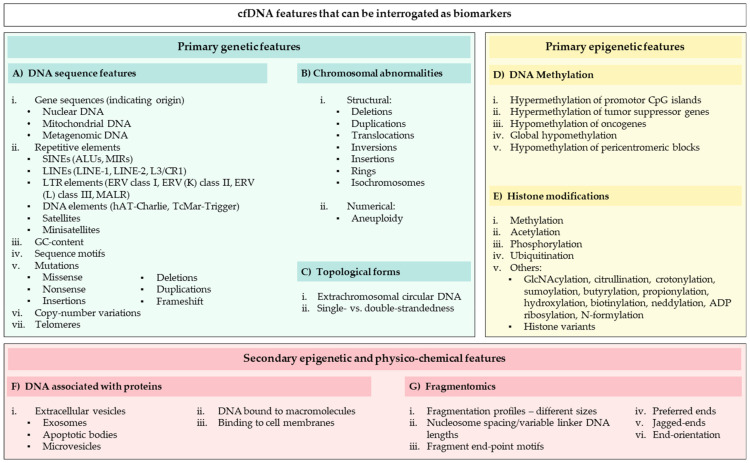
Features of cfDNA that could serve as clinical biomarkers. In addition to DNA hotspot mutations, various disease- and tissue-specific genetic, epigenetic, and structural features are encoded into cfDNA. Much of this information can be leveraged for the detection and monitoring of a wide range of diseases, physiological states, and other clinical scenarios.

Significant research efforts in the last decade uncovered a rich landscape of cfDNA physico-chemical features (reviewed in [130,137,138,139,140]). Beyond hotspot mutations, numerous cfDNA features are candidate biomarkers, including (i) various genetic features, such as DNA sequence features (Figure 4A), chromosomal abnormalities (Figure 4B), and topological forms (Figure 4C); (ii) primary epigenetic markers such as DNA methylation (Figure 4D) and histone modifications (Figure 4E); and (iii) various secondary epigenetic features that cfDNA molecules attain intracellularly or extracellularly following disruption of the primary structure of DNA, such as binding to proteins, extracellular vesicles, or cell membranes (Figure 4F), and fragmentomic features (Figure 4G).

One major advantage that most of the abovementioned features have over hotspot mutation profiling is that they occur across a large portion of the genome, which markedly increases their probability of detection. Indeed, the characterization of many of these markers are now considered as auxiliary tests to hotspot mutational profiling, while some epigenetic assays may even outperform mutational profiling and be used as stand-alone tests. Two breakthroughs are noteworthy here. First, the FDA-approved Epi proColon 2.0 CE test is routinely used to screen high-risk groups (patients over the age of fifty) for aberrant methylation of SEPT9, an indicator of colorectal cancer [141]. Second, a recent study used cfMeDIP-Seq to ID breast-cancer specific methylation signatures in the cfDNA of asymptomatic subjects, which allowed the detection of breast cancer several years before clinical presentation and diagnosis [142]. While they have not yet achieved the diagnostic sensitivity and specificity required for clinical implementation, numerous other studies have also demonstrated strong correlations between epigenetic features of cfDNA and wide-ranging disease activities, including various indications in different cancer types [143,144,145,146,147,148,149,150,151,152,153,154,155,156,157,158,159,160,161], CVD [162], liver damage [163,164], diabetes [165,166], multiple sclerosis [167], aging [51], and even psychological distress [168,169].

Another major advantage of epigenetic cfDNA features is that it may enable the parallel characterization of several cancer types. This is especially important in the context of cancer screening, where the identification of the tissue-of-origin of underlying cancers is crucial. This is a challenging task, but in the next section we explore how this is becoming increasingly possible through rapid progress in mapping various tissue-of-origin classifiers in the genome and in cfDNA [170,171].

### 5.1. Various Cell and Tissue-Specific Epigenetic Signatures in cfDNA

**Figure 5:**
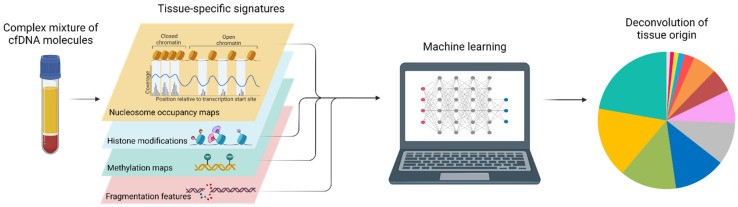
Determining the tissue-of-origin of cfDNA molecules. The cfDNA population in a typical biospecimen is highly complex and derives from numerous different cell and tissue types. However, cfDNA molecules contain multiple layers of cell-type specific epigenetic signatures, such as differentially methylated DNA regions, post-translational histone modifications, nucleosome occupancy, as well as various fragmentation features. Through the use of increasingly sophisticated molecular analysis methods coupled with machine learning algorithms, the epigenetic information carried by cfDNA molecules can be decoded to determine the contribution of different tissue types.

Cell-specific epigenetic features are conserved by cfDNA molecules and have been used for a variety of tissue-of-origin analyses in recent years. In particular, distinct approaches employing methylation patterns, nucleosome positioning, transcription factor binding site occupancies, and fragmentomic features were successfully developed to determine the tissue-of-origin of individual cfDNA molecules (Figure 5). Tissue-of-origin analysis enables estimation of the contributions of various tissues from the human body to the plasma DNA pool and thereby identify tissues where increased cell death occurs, potentially yielding sensitive screening tests for early diagnosis of a wide range of pathologies, therapy response monitoring, and detection of minimal residual disease.

#### 5.1.1. DNA Methylation

For methylation-based tissue-of-origin analyses, genome-wide methylation profiles of reference tissues (mostly based on whole genome bisulfite sequencing) were used to deconvolute cfDNA sequencing data. In 2015, a reference-based methylation deconvolution approach was developed to determine relative contributions of DNA from multiple tissue types to the plasma DNA pool [172]. This approach allowed the identification of differentially methylated regions unique to specific cell types, which enabled the researchers to identify the tissue-of-origin of cfDNA molecules in pregnancy, transplantation, and cancer. They also estimated that ≥70% of the plasma cfDNA was derived from white blood cells (i.e., neutrophils and lymphocytes) [172]. Considering the methylation state of a number of adjacent CpGs instead of a single CpG site significantly reduced background and enhanced the specificity of methylation-based tissue-of-origin analysis and was utilized to detect cell-type specific cell death from plasma samples with pathologies lacking genetic aberrations such as diabetes, multiple sclerosis, and head trauma [173]. Highly coordinated methylation sites, so-called methylation haplotype blocks, were the basis for another methylation-based tissue-of-origin method [174]. This approach investigated tightly coupled CpG sites and observed a reduction in completely coupled CpG pairs in cancer patients, which enabled quantitative estimation of tumor load and tissue-of-origin mapping in cfDNA of patients with lung or colorectal cancer [174]. Immunoprecipitation of methylated DNA followed by sequencing (cfMeDIP-seq) was developed specifically for low-input and already fragmented plasma DNA samples and enabled the classification of cancer in plasma samples from numerous tumor types [148]. Establishing reference methylation atlases of cell types (and not tissues) is very important to further advance methylation deconvolution approaches. One reference atlas of 25 human tissues and cell types covering major organs and cells involved in common diseases [92] and another recently published methylation atlas of 39 cell types sorted from healthy tissue samples [175] are available to further improve tissue-of-origin analyses via methylation patterns. The first commercially available blood-based multi-cancer early detection test was recently introduced by GRAIL [176,177,178], which utilizes targeted cfDNA bisulfite sequencing followed by ML and is able to detect cancer signals of multiple cancer types and predict cancer origin with high accuracy.

#### 5.1.2. Nucleosome Spacing and Occupancy

Many approaches have been developed to infer nucleosome positioning maps from cfDNA sequencing data. For this purpose, sequenced cfDNA fragments are aligned to the genome and genomic regions with higher number of fragments ending in those regions point to regions not occupied by a nucleosome, whereas regions that are spanned by the majority of sequenced cfDNA fragments are indicative of a genomic region protected by a nucleosome. One metric to quantify this is the windowed protection score (WPS) [179], which was used to determine the nucleosome spacing pattern that, in turn, informed on cfDNA tissues-of-origin. In healthy subjects, these nucleosome occupancy patterns matched hematopoietic lineages and additional contributions were detected in cancer [179]. Another approach employed whole genome sequencing of cfDNA in combination with nucleosome promoter analysis [180]. The read depth coverage at two discrete regions at transcription start sites was determined and enabled distinction of expressed and silent genes. The prediction of the expression status of individual genes can be employed for classification of expressed cancer driver genes [180]. The abovementioned WPS is also informative about the occupancy of transcription factor binding sites when determined for short fragments (35–80 bp) [179]. An alternative approach to assess transcription factor activity is based on cfDNA sequencing data and nucleosome footprint analysis [181]. A bioinformatics pipeline that infers accessibility of transcription factor binding sites from cfDNA fragmentation patterns has also been developed. By determining the activity of lineage-specific transcription factors, patient- and tumor-specific patterns were observed and allowed accurate prediction of tumor subtypes in prostate cancer [181].

#### 5.1.3. Histone Modifications

In one study, post-translational histone modifications have been successfully used for tissue-of-origin analysis. Chromatin immunoprecipitation and sequencing of cell-free nucleosomes directly from human plasma yielded information on DNA-related activities within the cells of origins [182]. Pathology-related changes in transcriptional programs in specific cell types could be identified with this method. In another proof-of-principle study, immunoprecipitation of H3 lysine 36 trimethylation (H3K36me3)-modified nucleosomes, a histone modification associated with active gene transcription, was used as a liquid biopsy marker to identify tumor-specific transcriptional activity of mutated alleles in non-small cell lung cancer [171,183].

#### 5.1.4. CfDNA Fragmentation Profiles

Fragmentomic features of cfDNA molecules can be used in various ways to determine tissue-of-origin and identify cell type contributors to the plasma DNA pool. First, cfDNA fragment size distribution can be employed for tissue-of-origin analysis. Selection for specific cfDNA fragments (90–150 bp) before sequencing enhanced the detection of ctDNA and identified differences in the size distribution of ctDNA and noncancer DNA fragments (i.e., ctDNA fragments are shorter than cfDNA from healthy samples) [184]. This ML-based method was able to detect multiple cancer types in plasma. A large-scale fragmentation pattern analysis approach evaluated cfDNA fragmentation profiles at megabase level across distinct cancer types [185]. This ML model incorporated genome-wide fragmentation features and could be used to identify the tissue-of-origin of multiple cancer types. Combining this fragmentation-based approach with mutation-based cfDNA analysis significantly enhanced detection [185]. Measuring the fragment length diversity at promoter regions of genes of interest via a targeted approach (EPIC-seq) and determining the promoter fragmentation entropy (PFE) allowed the inference of gene expression profiles and tissue-of-origin determination. The PFE approach was used to classify subtypes of lung carcinoma and diffuse large B cell lymphoma [186]. Second, the orientations of cfDNA fragments in open chromatin regions are informative of tissue-of-origin [187]. Determining the orientation in tissue-specific open chromatin regions where the respective tissues contributed DNA into the plasma allowed measurement of relative contributions of various tissues to the plasma DNA pool in pregnancy, organ transplantation, and cancer [187]. Third, the fragment end sequence motif (commonly the four bases at both ends of a cfDNA fragment) hold information about the cellular origins of cfDNA fragments. Analyzing the plasma DNA end motif via the motif diversity score (MDS, adopted from the normalized Shannon entropy) revealed distinct characteristic sets of plasma DNA end motifs for plasma DNA molecules derived from liver, hepatocellular carcinoma and other cancers, and placenta and hematopoietic cells [188]. A significant increase in the diversity of plasma DNA end motifs in patients with hepatocellular carcinoma was observed, aberrant end motifs were also found in patients with other cancer types [188]. A genome-wide catalogue of cfDNA fragment end sequence patterns of patients with 18 different cancer types demonstrated enhanced cancer detection, monitoring and prognosis [189]. Here, the authors calculated the normalized Shannon index and a Gini index of the 5′trinucleotide and the 5′ and 3′mononucleotide, respectively, to quantify the extent of cfDNA fragment end diversity and demonstrated that the so-called Fragment End Integrated Analysis (FrEIA) score could be employed to quantitatively evaluate the tumor fraction in plasma samples.

While the clinical utility of most of the abovementioned cfDNA features still need to be evaluated and confirmed, sufficient evidence indicates that their analysis may expedite the development of increasingly powerful, clinically meaningful cfDNA-based assays. However, similar to the problems encountered in the development of hotspot mutation-based cfDNA tests, epigenetic profiling of cfDNA is challenged by numerous biological, preanalytical, technical, and analytical issues and limitations. Among many, some of the most significant challenges are (i) similar or identical modifications that arise in both ordinary biological processes and pathologies, (ii) stochastic fluctuations in epigenetics or specific epigenetic biomarkers can induce significant biological noise, and (iii) various biases of different methods can alter the observed epigenetic profile and complicate accurate analysis (reviewed in ref. [140]). To develop clinically meaningful assays, these challenges need to be overcome.

## 6. Other Sources of Cell-Free DNA

The bulk of cfDNA research to-date is centered on the characterization of DNA originating form the nucleus through cell death. However, increasing evidence indicates that various other sources such as mitochondrial DNA, neutrophil extracellular traps, and extracellular vesicles, which may also originate from non-apoptotic processes, may have significant clinical utility.

### 6.1. Mitochondrial DNA (mtDNA)

Mitochondria are intracellular organelles that perform a variety of essential functions. They are the primary generators of cellular energy, produce several biosynthetic intermediates (e.g., haem, lipids, and amino acids), and are involved in cellular stress responses, including apoptosis, innate immunity, and hypoxia. Apart from its cell autonomous roles, mitochondria can also influence an organism’s physiology by regulating intercellular and interorgan communication [190,191,192]. Thus, considering its central role in human physiology, it is not surprising that mitochondrial dysfunction is associated with numerous disorders, and that cell-free mitochondrial DNA (cf-mtDNA) is attracting increasing attention as a potentially versatile biomarker for a wide range of diseases and other clinical scenarios. The presence of cf-mtDNA in the human circulatory system was first reported in a study in which a known mtDNA mutation was identified in the DNA isolated from the plasma and serum of type 2 diabetes mellitus patients [193]. Since then, numerous studies have confirmed the presence of cf-mtDNA in the circulation of both healthy and diseased individuals [194].

For example, aberrant cf-mtDNA has been correlated with various cancers [195,196,197], including breast cancer [198], lung adenocarcinoma [199], squamous cell carcinoma [200,201], Ewing’s sarcoma [202], urological malignancies [203], and oral cancer [204]; neurodegenerative diseases [205] such as Friedrich’s ataxia [206], multiple sclerosis [207], and Parkinson’s disease [208]; diabetes [193,209]; aging [210]; surgery and trauma [211,212]; sepsis [213,214]; HIV [215,216,217] and type-2 diabetes [218]-associated cognitive decline and chronic inflammation; exposure to carcinogenic pesticides [219]; adverse effects of spaceflight on the health of astronauts [220,221]; poor outcomes of patients with adult community-acquired bacterial meningitis [222]; psychological issues such as major depressive disorders [223], suicidal behavior [224], and acute psychological stress [225], as well as psychosocial and physical stress [168]. Reports have also shown that cf-mtDNA characteristics can be influenced by exercise [226,227], suggesting a possible use in sports medicine. Another potential use of cf-mtDNA may be minimally invasive haplogroup matching [228].

Cf-mtDNA may also have underappreciated potential as a biomarker for cardiovascular disease (CVD), which is one of the leading causes of death worldwide [229,230,231]. MtDNA aberrations on the level of mutations, copy number shifts, and methylation changes are associated with a wide range of CVDs [232,233,234,235]. Prolonged reduction of blood flow to the heart, as a result of atherosclerosis, for example, decreases the amount of oxygen available for the mitochondria of cardiomyocytes in the territory of the blocked artery. This results in the rapid depletion of cellular ATP, triggering an ischemic cascade, and eventually the induction of cell death through apoptosis or necrosis. If tissue death is severe enough, this can lead to a myocardial infarction (MI) [236,237]. Since mtDNA is released into the bloodstream during this process, cf-mtDNA levels may serve as a potential biomarker for estimating the course and outcome of injury in patients following MI. A number of studies have demonstrated significantly elevated levels of cf-mtDNA in patients with MI compared with healthy subjects [238,239,240,241], and cf-mtDNA levels appear to correlate with the degree of myocardial damage and are also higher in transmural MI than in non-transmural MI [238]. Whether cf-mtDNA levels correlate with MRI infarct size is not yet known. Furthermore, cf-mtDNA levels in MI patients decline significantly following percutaneous coronary intervention treatment [239,242]. Apart from MI, elevated levels of cf-mtDNA have also been demonstrated in patients with chronic systolic heart failure [243] and sudden cardiac arrest compared with healthy control subjects and survivors [231]. Furthermore, while there is a paucity of publications on this subject, no study has yet demonstrated a clear correlation between cf-mtDNA levels and most vascular risk factors, such as hypertension, dyslipidemia, and smoking status. However, studies have indicated that cf-mtDNA is significantly increased in patients with diabetes mellitus (DM) [244], and is higher in CVD patients with DM than in CVD patients without DM [245].

MtDNA can be released into body fluids through various modes of cell death [1,246], or by regulated processes, where certain mechanisms actively secrete cf-mtDNA via mitochondria-derived vesicles and neutrophil extracellular traps (NETs). Many cell types such as leukocytes, endothelial cells and platelets, seem to be able to release mtDNA [247], while it was recently also shown that intact respiratory-competent mitochondria circulate in blood plasma, which may also serve as a source of cf-mtDNA [248]. The structure of cf-mtDNA remains poorly characterized; however, it seems that it can be present either in naked form or associated with internal and external mitochondrial membrane fragments [249]. Furthermore, the absence of nucleosome-associated histone proteins and, therefore, the absence of higher-order packaging of mtDNA render “naked” cf-mtDNA exposed to enzymatic cleavage. This suggests that cf-mtDNA molecules should be more fragmented than autosomal cfDNA. Indeed, unlike autosomal cfDNA, which is typically characterized by a three-mode size signature representing apoptotically generated mono-, di-, and tri-nucleosomes, cf-mtDNA generally exhibits a wider range of sizes. Some studies have shown that cf-mtDNA is highly enriched in fragments ranging between 40–80 bp [106,246], while others have observed a broader size distribution of 50–300 bp [250,251,252]. An important point to bear in mind is that the aforementioned studies have used different methods of cf-mtDNA purification (e.g., QIAamp DSP DNA Blood Mini Kit vs. DNeasy Blood and Tissue Kit) and fragment sizing (e.g., paired-end sequencing vs. qPCR using specific amplicons). Plasma cfDNA obtained with non-hybridization-based extraction methods, which do not filter and capture DNA fragments of a specific size, have revealed very different size profiles [61]. Moreover, since a fraction of cf-mtDNA fragments can be associated with particles [249], the characteristics of extracted cf-mtDNA can be influenced by other preanalytical processing steps, such as centrifugation. It is therefore possible that these differences in reported cf-mtDNA sizes are merely an artefact of variable methodology and may not be a true reflection of their in vivo counterpart. It is thus clear that there is insufficient data to reach a consensus on the size and exact structure of cf-mtDNA. In order to gain a better understanding, it is necessary to establish a standardized blood processing method and to perform a systematic characterization of both cf-mtDNA and cf-nDNA, perhaps making use of in vitro cell cultures, well established animal models, or samples collected from large clinical cohorts [225,253].

### 6.2. Neutrophil Extracellular Traps (NETs)

Neutrophils are the most abundant innate immune effector cells, accounting for over 50–60% of white blood cells. Since NETs are composed of a DNA scaffold, they are considered to be important sources of circulating cfDNA [254]. Extracellular traps (ETs) produced by neutrophils were first observed in 2004 [255] and to date it has been shown that other immune cells including macrophages, mast cells, eosinophils, basophils, plasmacytoid dendritic cells, and lymphocytes are all able to produce ETs [256,257,258,259]. As neutrophils are present in significant amounts in blood, we will focus on NETs in this review.

**Figure 6:**
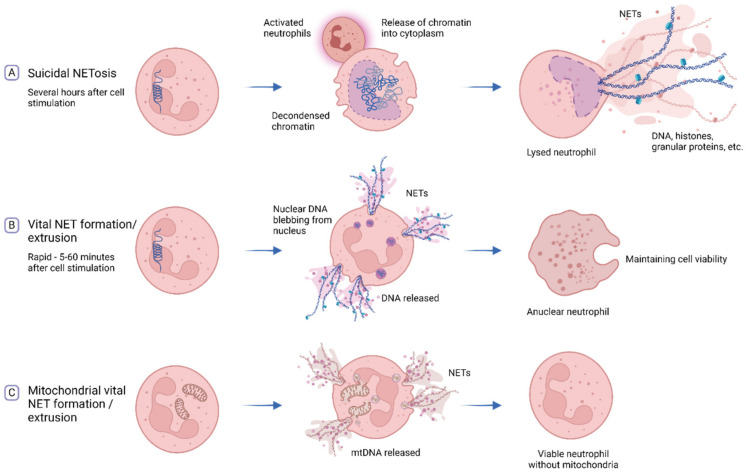
Mechanisms of NET formation. (**A**) Suicidal NETosis occurs over several hours and involves the decondensation of chromatin in activated neutrophils leading to the release of chromatin into the cytoplasm. Subsequently, the cell membrane is ruptured and chromatin is released from the lysed neutrophil into extracellular space. (**B**) Vital NET formation/extrusion is a rapid process occurring within 60 min of cell stimulation where vesicles containing nuclear DNA are fused with the plasma membrane and chromatin is released entirely, leaving anuclear neutrophils. These cells maintain their viability; therefore, this type of NET formation is best categorized as NET extrusion rather than NETosis, which implies that the NET-forming cells are lysed. (**C**) Mitochondrial vital NET formation/extrusion is a secondary form of vital NET formation in which mitochondrial DNA is released as NETs after the rupture of mitochondrial membranes, leaving viable neutrophils lacking mitochondria.

NETs consist of a meshwork of DNA fibers bound to histones and cytoplasmic and granular proteins [260]. They are described as three-dimensional net-like [261] or cloud-like [262,263] structures, with differences possibly resulting from varying DNA origins, formation mechanisms, or experimental settings [261,263,264]. Both mitochondrial [265,266] and nuclear DNA [262] have been found to form or at least make up parts of NETs. As reviewed elsewhere [267,268,269], the details of the different pathways involved in NET formation is still rather controversial and highly debated. With this in mind, we briefly describe what is known to date about the mechanisms of formation (Figure 6). Two principal mechanisms of NET formation have been described, namely (i) suicidal NET formation, or NETosis, which is a unique form of cell death that lasts several hours [270]. After neutrophil activation, nuclear chromatin decondenses and expands, followed by disassembly of nuclear and granule membranes and release of chromatin into cytoplasm, where cytoplasmic and granule proteins are bound [270]. Finally, the plasma membrane disrupts, and the decorated DNA filaments are released. Additionally, mtDNA is released into cytoplasm via a controlled pore forming mechanism during apoptosis. It is unclear whether this is contributing to NET formation [271]; (ii) Vital NET formation or extrusion is a rapid process occurring within 5–60 min. Here it is important to note that these cells fully maintain their viability, and therefore this type of NET formation is described as NET extrusion rather that NETosis, which implies the lysis of the NET-forming cells [272]. Both nuclear and mtDNA have been described as components of these types of ETs [265,273]. Nuclear DNA is released in vesicles by blebbing directly from the nucleus while the nuclear and plasma membrane initially remain intact [273,274]. The nuclear membrane may subsequently rupture, leading to an accumulation of chromatin fibers into the cytosol, while retaining their viability. In 2009 Yousefi et al. demonstrated that NETs from viable neutrophils can also be composed of mtDNA. Two modes of mitochondrial NET formation/extrusion have been suggested so far: (a) mtDNA may be released into cytosol where it is further packed into vesicles and released by fusion with the plasma membrane thereby forming traps [275]; (b) The direct release of mtDNA and granules in a catapult-like manner via fusion of the mitochondrial and plasma membrane [269,271,275].

Compared with NET formation, much less is known about their degradation, although the imbalance between the formation and degradation of NETs is relevant to the development of various diseases. It has been shown that DNase I can disrupt the NETs scaffold in vitro [276], but with physiological concentrations of DNase I, it is not capable of fully digesting it. There is evidence that DNase I preprocesses NETs extracellularly, which are then cleared by macrophages [277]. Recently it was shown that proinflammatory polarized macrophages have an increased uptake and boosted further macropinocytosis. Additionally, macrophages secrete DNases, e.g., DNase I and DNase 1L3, which disrupt NETs in preparation for intracellular uptake [278]. DNase 1L3, released by dendritic cells was also found to take part in the extracellular degradation, whereas TREX1 (DNase III) was found to degrade NETs intracellularly within macrophages [279]. In case of insufficient uptake by macrophages, it is possible that partially digested DNA fragments are released. Another factor which contributes to the degradation through macrophages is complement component 1q (C1q), as it has been reported to facilitate the process by opsonizating NETs [277]. There is an ongoing discussion as to whether the antimicrobial peptide LL-37 from neutrophils facilitates the uptake into cells and protects them against degradation by bacterial and cellular nucleases [279]. Overall, additional studies are still needed to identify other nucleases that are potentially involved in NET degradation and to further characterize the mechanisms of extracellular DNA uptake into cells.

### 6.3. Extracellular Vesicles (EVs)

Extracellular vesicles (EVs) are lipid-bound vesicles that are naturally released by almost all cell types into extracellular space and have been found in all biological fluids tested thus far [280,281,282,283,284,285]. The different types of EVs are classified according to their biogenesis, content, function, and size (Table 2). Several in-depth reviews have described the biological characteristics and functions of EVs [281,286,287,288,289]. Here our primary focus is to review evidence of EVs as a source of cfDNA.

The most extensively studied small EVs (sEVs) are exosomes. They range in size from 40 to 100 nm and are constitutively generated from late endosomes [290,291,292,293]. Numerous studies have reported the presence of single- and double-stranded DNA as well as mtDNA [294,295,296], transposable elements [297] and viral DNA [298,299] in association with sEVs. The primary localization of exosomal DNA is not clear, as studies have shown DNA to be located in the lumen and on the outer surface [300,301,302]. Interestingly, a recent study reported that exercise triggered an increase of DNA levels only on the outer surface [301]. While there is ample evidence of exosome-associated DNA, it remains a controversial topic as highly cited studies have reported evidence both for the association of DNA with exosomes [295,303,304] and no association of DNA with exosomes [305]. Apart from biological diversity among different exosome types, which are yet to be fully explored, there may be other reasons for the discrepant findings reported by studies, such as differences in sample handling and exosome isolation protocols [306]. Furthermore, despite the efforts by the International Society of Extracellular Vesicles to provide guidelines for conducting sEV experiments (MISEV2018) [289], it is often the case that simple requirements such as the reporting of the volume of starting material, volume of sEV isolated or number of isolated EVs used for DNA isolation are not met by many publications. This makes it very difficult to reproduce experimental findings or to theorize about possible structures and mechanisms. Other methodological hurdles may include Western blots that lack a size marker, whether reducing conditions were used [298,307,308], or whether fetal bovine serum was included in cell culture experiments [309]. These factors are important because in the context of biofluids the presence of CD81 and CD9 can overlap with human low chain IgG cross-reactivity with the secondary antibody (see [307,310] for light and heavy chain IgG bands) unless non-reducing conditions are used or highly cross-adsorbed secondary antibodies. Despite controversial reports, conflicting data, and an array of preanalytical and analytical hurdles, a growing body of evidence indicates that sEV-DNA may have diagnostic uses and also play an important role in biological and pathological processes. For example, sEV-DNA may serve as cancer biomarkers [309,311,312,313], act as intercellular messengers [297,314,315,316], and play a role as a mediator of proinflammatory effects [317,318] and viral responses [298,299,319].

Gram-negative bacteria release outer membrane vesicles (OMVs), which are sEVs (20–250 nm) released from the outer membrane that encapsulate periplasmic contents. Their role and potential are extensively reviewed in ref. [320]. There is evidence that these OMVs can cross the intestinal epithelial barrier and reach the bloodstream where their DNA content have been detected in healthy individuals [320].

ARMMs are a type of sEV (40–100 nm) that bud directly from the plasma membrane and have a size similar to that of exosomes [321]. To our knowledge, ARMMs are the only class of EVs that have not yet been shown to be associated with DNA. However, it is interesting to note that ARMMs are likely to be co-purified with exosomes through most sEV isolation methods due to their similar size and the fact that they also contain the transmembrane tetraspanin CD9.

Microvesicles (MVs) are the most diverse EV population with regard to their size (0.1–1 µm) and they are generated via direct outward budding of the plasma membrane [322]. Tumor-derived MVs (TMVs) have been shown to contain dsDNA and they could potentially play a role in inhibiting tumor progression [323]. An increase of MVs and dsDNA in MVs is reported in relation to lupus nephritis [322].

Large oncosomes are large EVs (1–10 µm) secreted exclusively by cancer cells [324], and are reported to contain more DNA than sEVs and their DNA cargo includes tumor-specific alterations [308].

Apoptotic bodies (ApoBDs) are the largest of the EVs (0.5–2 µm) and are released during apoptosis [325,326]. They contain a wide variety of cellular components including micronuclei and DNA fragments. When not digested by DNASE1L3, ApoBD-associated DNA can bind directly to antinuclear antibodies, a common feature of autoimmune conditions such as systemic lupus erythematosus [327].

Exomeres are novel extracellular particles (35–50 nm) discovered with the use of asymmetric flow field-flow fractionation (AF4) and although they are not vesicles, we decided to include them as they have been shown to complex with DNA. They are found to be associated with large fragment DNA but their biogenesis, function, and potential in diagnostic, prognostic, and therapeutic applications remain to be elucidated [328].

In summary, all EVs apart from ARMMs have been reported to be associated with DNA [300,308,322,323,327,328,329], which may have significant implications for the cfDNA research field and currently represents not only an underappreciated source of cfDNA, but also a potentially distinct and biologically active subpopulation of cfDNA. However, there are still many unanswered questions, conflicting data, and obstacles related to the phenomenon of EV-associated DNA that need to be resolved. One major unknown is that it is still unclear what percentage of the total circulating cfDNA population is associated with EVs. This number has, for example, been reported to be as little as 5% [330] and as high as 90% [310]. In line with this, one major obstacle that needs to be addressed is the lack of standardization of methods. A likely reason for the significant difference in the values reported by the two aforementioned studies, for example, may be the use of different sample handling procedures. In one study samples were processed immediately after blood draw [330], whereas samples used in the other study were only processed at a later time [310]. Given the short half-life of unbound cfDNA [75,76,77], such a delay could significantly impact cfDNA yield and integrity [7]. Other important points that remain to be clarified are (i) the relative contribution of different EV types toward the total cfDNA population in general and specific cases, (ii) whether EV-associated DNA is packaged mainly within the interior of these vesicles or if they are mainly adhered to the exterior surfaces; (iii) the relative proportions of the total EV-associated cfDNA in both these locations; (iv) the mechanisms involved in the binding of cfDNA fragments to the EV surfaces of different types of vesicles; and (v) the effect of other physiological and biological factors that control these mechanisms [254].

## 7. Multimodal Analysis, Data Integration, and Machine Learning (ML)

Evidence suggests that the sensitivity and specificity of cfDNA assays may not only be augmented by the simultaneous interrogation of multiple cfDNA features, but also by the parallel characterization of cfDNA and other biomarkers, such as proteins [331,332], genomic DNA and RNA from circulating tumor cells (CTCs) [333,334,335,336,337,338,339,340], EVs [338,341], miRNAs [342,343], metabolites [344], or mRNA transcripts [345].

Due to the sheer amount of data points generated per sample by the characterization of multiple cfDNA features or multiple different biomarkers, the trend in recent years has been to extract a “signature” from multiple biomarkers instead of focusing on individual data points. This necessitates a radical change to the traditional way of data analysis and interpretation, and mainly involves the application of ML. Indeed, an increasing number of cfDNA studies are using ML with great success [154,158,175,180,184,185,332,346,347,348,349,350,351,352,353,354,355,356,357]. ML is usually accomplished through pre-processing, featurisation, feature selection, model fitting, and evaluation. ML methods range from logistic regression, random forest, and support vector machines to neural networks including their counterparts, deep neural networks. ML methods can capture sophisticated signatures, but as model complexity increases, it also becomes more difficult to interpret data. This is related to the “curse of dimensionality”, which increases with the number of features in the ML model. This can be particularly problematic when there are more features than samples in the data set. To make interpretation easier, the number of samples in a data set can be increased. For instance: Deep neural networks can detect signatures in samples with many features, such as flow cytometry images [81], but there is a high probability of overfitting if the number of available samples is less than the number of features used. This configuration is frequent in cfDNA experiments and is one motivation for performing extensive feature selection to incorporate only the most informative bits of each sample. For example, the construction of a reference atlas of tissue samples is often based on selected regions that distinguish a particular tissue from other tissues. One group used fewer than one hundred features per tissue as a reference [175]. This procedure is also known as dimensionality reduction and is performed, e.g., in neural networks or support vector machines. However, even if these precautions are taken, larger cohorts and other mechanisms are still needed to ensure interpretability and generalization of the model, where the latter refers to the ability to find the relevant signals in new samples. Another method to avoid overfitting is regularization, which penalizes overfitting and thus makes the model less specific. Finally, data augmentation can help to artificially increase the sample size by creating new samples from previous samples.

Along with the data processing problems comes the possibility of finding signatures obscured by interacting markers that were not previously visible. In addition, this gives researchers the opportunity to conduct research with shallower sequencing depths. Databases that contain references help researchers to select regions of interest more specifically, and also provide the opportunity to test their algorithms on other datasets and infer different epigenetic features or omics [180]. Table 3 provides an overview of some recent cfDNA papers describing ML methods used for their main objective.

## 8. Potential Pathological Effects and Biological Functions of cfDNA

As discussed in Section 2, elevated total cfDNA levels have been correlated with a wide range of clinico-pathological data, but an overflow of overlapping variables renders the assessment of total cfDNA inadequate as a stand-alone biomarker. However, the phenomenon of elevated cfDNA levels may be more biologically relevant than previously thought. Many early studies and a growing body of new work indicate an underestimated role of different cfDNA types (e.g., cf-nDNA, cf-mtDNA, NETs, and EV-DNA) in the development and progression of various diseases. Moreover, apart from detrimental effects, several studies and some elegant theoretical work suggests a possible role for cfDNA molecules in immunomodulation, the maintenance of cellular homeostasis, as a mode of intercellular communication, and underappreciated factor in evolutionary processes. In the following section we explore the various detrimental effects and possible biological functions of cfDNA molecules, and the pathways through which they may elicit its effects.

### 8.1. Oncogenesis, Cancer Progression, and Metastasis

There is some evidence implicating cfDNA in oncogenic transformation, tumor progression, and development of metastases [361,362,363]. While the molecular mechanisms and cellular circuits by which cfDNA mediate these observed tumor-promoting effects remain mostly unknown, several reports indicate that the lateral transfer of cfDNA between cancer cells and neighboring or distal cells may be sufficient to propagate the genetic alterations required for transformation, and at the same time activate signaling pathways that promote malignant growth. CfDNA or fragmented chromatin from cancer cells have been shown to readily infiltrate surrounding cells and incorporate into host genomes, inducing double-stranded DNA breaks (DSBs), genome instability, cell death, inflammation, and destabilization of the homeostatic capacity of the tumor microenvironment, all of which are potent stimuli for oncogenic transformation and tumor progression [114,364]. The lateral transfer of overexpressed therapy-augmented cfDNA molecules, such as oxidized DNA [365,366,367,368] or duplicated oncogenes [369,370], can cause accelerated DNA damage, thereby conferring resistance against radio- and chemotherapy. In line with this, there is evidence that cfDNA released by dead or dying tumor cells can activate TLR9 signaling, which in turn inhibits apoptosis and enhances autophagy, thereby promoting tumor growth [371]. Furthermore, it is possible that cfDNA promotes metastasis in recipient cells by inducing the overexpression of several pro-metastatic genes, which for example enhance cell invasion and migration, through the TLR9/MYD88 independent pathway [365,372,373], while cfDNA may also promote metastasis by altering the expression levels of the inflammatory chemokine CXCL8 [374], or other genes such as MMP9 and CD44 and miRNAs [375]. Other reports suggest that the malignant phenotype of tumor cells may be transferred to neighboring normal cells via the assimilation and transfection of cfDNA and other nucleic acids complexed with EVs, such as apoptotic bodies [326] and exosomes [291,376]. It is not yet clear how cfDNA fragments are integrated into host genomes, but some evidence suggests that it may occur through the non-homologous end-joining (NHEJ) double-strand DNA-repair pathway [113,364,377]. Alternatively, ctDNA enriched in specific retrotransposons (e.g., a hot LINE-1 element) [139,378,379] could, by virtue of their inherent mobility, penetrate recipient cells and initiate tumor formation upon “cut-and-paste” insertion into host tumor suppressor genes [380,381]. While there is no direct evidence, it is interesting to speculate here on the possibility of lateral transfer of therapy resistance mutations from resistant cells to surrounding cells.

In contrast to pro-cancer effects, it has also been demonstrated that the lateral transfer of cfDNA derived from healthy cells can halt the proliferation of cancer cells [382,383]. An early study showed that mouse-derived tumor cell lines cultured in growth medium supplemented with cytosol from non-dividing lymphocytes or hepatocytes was mirrored by a decreased rate of [3H]-thymidine incorporation and inhibition of tumor growth. Despite the molecular complexity of cytosol, this inhibitory effect was ascribed to the DNA present in the cytosol of the lymphocytes or hepatocytes [384]. More recently, in vitro [383] and in vivo [382] studies have shown that cfDNA from healthy cells can be used to disrupt DNA synthesis in dividing tumors, thereby hampering or completely blocking tumor growth and preventing metastasis. One study also showed that water-soluble nuclear crude extract prepared from salmon soft roe could inhibit the growth of both cancer and non-cancer cell lines through delaying cell cycle progression [385]. While the use of cfDNA as an anti-cancer agent may be an intriguing approach, the research is currently scant, and more evidence is needed. Alternatively, if sufficient evidence indicates the tumor-promoting effects of cfDNA, direct targeting and elimination of cfDNA may constitute a potential therapeutic approach. For example, non-specific degradation of cfDNA in pancreatic cancer cell cultures via DNase I resulted in a reduction of matrix attachment, migration, and invasion, thereby reducing metastatic potential [374]. Similarly, in an in vivo orthotopic xenograft model, DNase I treatment alone suppressed tumor metastasis [374]. Given the uncertainties related to the potential effects or consequences of untargeted or nonspecific DNA digestion in cancer patients, it is interesting to note here that cationic nanoparticles have been successfully used for scavenging cfDNA in a rheumatoid arthritis rat model, which resulted in the inhibition of inflammation and relief of symptoms [386].

### 8.2. Functions and Effects of NETs

A wide range of bacteria, fungi, parasites, and viruses can induce the formation of NETs (reviewed in refs. [267,387]). Thereby, pathogens can be immobilized, and their elimination is facilitated by antimicrobial compounds and phagocytosis. Whether NETs themselves are capable of killing microorganisms in vivo is not yet clear, but since many of their components are toxic to microorganisms, it might be possible [388,389]. NETs and their components are rapid danger signals, and thus activate necessary immune responses. On the other hand, evidence is accumulating that they are able to amplify inflammation and trigger the formation of additional NETs, which contribute to direct tissue damage, particularly via histones and proteases [267,268,390]. Their possible associations with various diseases are reviewed in detail elsewhere [275,390,391]. Among others, they are described in relation to severe sepsis, cystic fibrosis, asthma, and CVDs, including thrombosis, systemic sclerosis, myocardial infarction, and stroke. In addition, DNA, histones and neutrophil proteins can trigger the production of autoantibodies. This could contribute to the clinical features of SLE, RA, and other autoimmune diseases. There are also indications that NET-derived granule proteins might contribute to the migration of tumor cells [390].

The components of NETs are widely discussed as potential biomarkers and have recently received additional attention due to their correlations with coronavirus disease 2019 (COVID-19). Levels of circulating myeloperoxidase (MPO)-DNA complexes, a marker of NETs levels, were observed to be increased in symptomatic COVID-19 patients. Circulating NETs are thought to be implicated in the pathology of COVID-19 by causing capillary destruction and leakage, and by inducing endothelial cell death and thrombosis. These circumstances can lead to lung epithelial destruction and death [392]. Thus, it has been suggested that the MPO-DNA complex should be further investigated as a biomarker for the early phase of severe acute respiratory syndrome coronavirus type 2 (SARS-CoV-2) infection [393,394]. NETs could also potentially serve as biomarkers in other diseases, such as cancer. In a recent study, researchers were able to distinguish between patients with metastatic colorectal cancer and healthy individuals based on cfDNA, MPO, and neutrophil elastase (NE) concentrations. It was further suggested that cfDNA levels, in combination with MPO-DNA and NE, could serve as potential biomarkers for disease severity, prognosis or treatment guidance of metastatic colorectal cancer and cancer in general [395]. Furthermore, correlation of the markers mentioned above with levels of autoantibodies, e.g., anti-dsDNA, may give insight into autoimmune activity [396].

Not only may NETs and their components serve as biomarkers in the elucidation of diseases, but, due to their involvement in pathogenesis, they also represent potential therapeutic targets. For example: (i) In preclinical studies, targeting NADPH-dependent chromatin decondensation by ROS scavengers, like N-acetyl cysteine, MPO inhibitors and PAD4 inhibitors, was shown to suppress NET release [268,397]; (ii) An additional target was recently highlighted by Cao et al. They demonstrated an efficient inhibition of NET formation in vitro by a DEK-targeting aptamer (DTA). DEK is a chromatin-associated protein which may play a crucial role in the formation of NETs and serves as an autoantigen in many autoimmune diseases. In addition, they modified DTA chemically and improved its application and achieved an attenuated inflammation in collagen-induced arthritis mice [398]; (iii) another interesting therapeutic approach was proposed by Chiang et. al. [399]. They investigated specialized pro-resolving mediators (SPMs) and encountered a new series that appear to be potent agonists of pro-resolving phagocyte functions. The resolvins, termed 13-series (T-series) resolvins (RvTs), are produced by human cells and blood. Chiang et al. prepared RvTs by total organic synthesis and found a reduction of NETs in PMA-stimulated human whole blood. Furthermore, they found a potent structure of RvTs which activated the cAMP-PKA-AMPK pathway in human macrophages, resulting in improved NET clearance [399]; (iv) Treatment with DNases is an alternative way to accelerate the degradation of NETs. Dornase alpha, a recombinant human DNase I, is already in use for treatment of cystic fibrosis and was tested in a clinical trial for the treatment of acute respiratory distress syndrome secondary to COVID-19. During drug administration, a reduction of MPO-DNA complexes in bronchoalveolar lavage fluid and improved oxygenation and lung compliance were observed. This again illustrates the potential role of NETs in the pathology of COVID-19 [400]. There may be many more approaches to target NETs, such as direct inhibition of granular proteins and enzymes. However, it is necessary to evaluate them not only in vitro but also in vivo and to investigate the more specific pathways of formation in both diseased and healthy conditions.

In summary, while much is still not known about the biology of NETs, it clearly represents an important and biologically active class of cfDNA molecules that deserves more research attention.

### 8.3. Other Potential Functions of cfDNA

Several studies have shown that cfDNA molecules can enter recipient cells and elicit a variety of biological responses, either by integrating into the host genome or by other intracellular activities [4,113,401]. This phenomenon has not only been implicated in the detrimental processes described above, but has also been implicated in benign intercellular messaging functions, such as the synchronization of cell differentiation [4], as well as evolutionary processes, such as the lateral transfer of hereditary information from somatic cells to germ cells or adaptive traits between different cells (reviewed in refs. [5,402,403]). However, the possibility of cfDNA as an intercellular messenger or a factor in evolutionary processes has not yet been taken seriously by the research community.

While it is true that there currently lacks a concrete body of evidence for these functions of cfDNA, it is also true that the research field is significantly biased toward clinically motivated studies, and that there is a general lack of interest in cfDNA as a biological phenomenon in general. Yet there seems to be sufficient evidence to warrant further enquiry into the possible biological functions of cfDNA. A more detailed and structured enquiry into the biological functions of cfDNA may reveal currently unknown processes involved in the mechanisms underlying various pathologies. In line with this, targeted elimination of aberrant cfDNA molecules may represent a potential therapeutic strategy in a variety of pathological scenarios.

## 9. Biological Factors That Affect cfDNA Measurements

**Figure 7:**
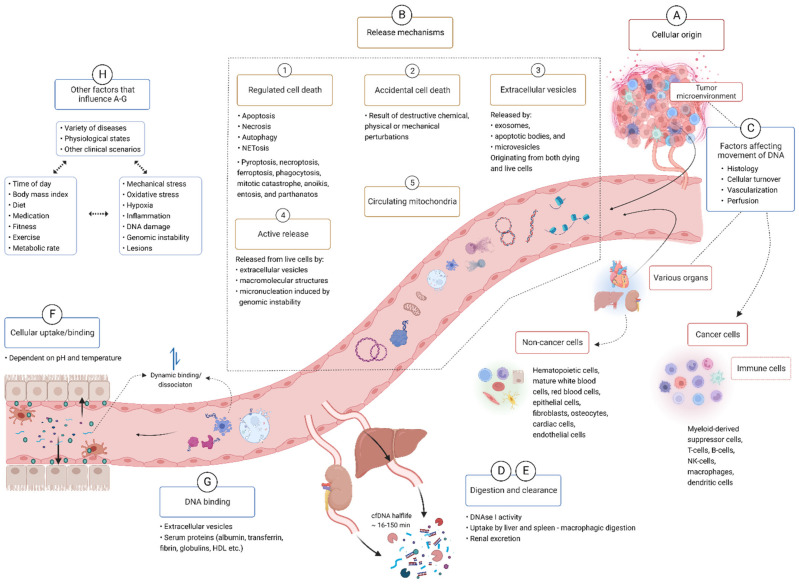
Biological factors that modulate the characteristics of cfDNA. The composition and fluctuation of the cfDNA population in the circulatory system is influenced by numerous, often mutually non-exclusive determinants. The major factors include (**A**) the relative contribution that different cell types make toward the total cfDNA pool; (**B**) the mechanisms by which cfDNA is released from the various contributing cells; (**C**) factors that affect the movement of cfDNA from tissues or cells into circulation; (**D**) modifications and rate of degradation by extracellular nucleases and proteolytic enzymes; (**E**) the rate of uptake and digestion by the liver, spleen or kidneys; (**F**) binding and detachment to circulating or epithelial cells, and (**G**) association with DNA-binding proteins, other macromolecules, or extracellular vesicles. (**H**) All of the former factors are amendable by a web of factors, many of which may interact in known and unknown ways, including (i) a variety of diseases, physiological states, and other clinical scenarios, (ii) phenomena that cause cell death or constitutive DNA release, such as mechanical stress, oxidative stress, hypoxia, inflammation, DNA damage, genomic instability, lesions, and (iii) other factors such as, e.g., time of day, body mass index, diet, medication, fitness, exercise, and metabolic rate, most of which are liable to significant intra- and interindividual variation.

Early studies have focused mainly on the characterization of circulating cfDNA (i.e., blood plasma or serum), but cfDNA molecules have now been detected and are increasingly investigated in all non-circulatory body fluids (e.g., urine, saliva, stool). The composition and fluctuation of cfDNA are modulated by a variety of factors, many of which are unique to or vary depending on the characteristics of specific body fluids. While there are some overlapping factors among the different body fluids, we limit the focus here to an exploration of the most prominent biological factors that affect the quantitative and qualitative characteristics of cfDNA in relation to its analysis in blood plasma (Figure 7).

### 9.1. Relative Contribution of Different Genomes

While it shifts depending on the presence of disease and many other factors, the relative percentage contribution that different origins of cfDNA (e.g., DNA derived from different host-cell types, nuclei, mitochondria, endogenous microbes, pathogens, etc.) make toward the total cfDNA pool has not yet been determined in healthy individuals or in specific pathological or physiological states. However, in cancer patients it is, for example, well understood that the respective contributions of wild-type vs. tumor-derived DNA depends on cancer stage but exhibits significant intra- and interindividual variation [9,404]. Moreover, regarding wild-type cfDNA of host-cell origin, there is no scientific consensus on the relative percentage contributions of various tissue and cell types, but there are two important observations. First, several studies indicate that the majority of cfDNA originates from hematopoietic cells [173,405,406] and mature white blood cells [172,407,408,409]. Second, despite a major contribution from blood cells, cfDNA has been found to originate from many different cell types with significant heterogeneity in the relative contributions observed among different studies [81]. A better understanding of this variation can only be gained through increasingly accurate estimations of the cellular origin of cfDNA, facilitated by (i) methods that allow total cfDNA extraction and (ii) studies that systematically map the landscape of cell- and tissue specific features of cfDNA [92,174,179,347,348,354,410,411,412,413].

### 9.2. Mechanisms of Release and Intracellular Modifications

Apoptosis is normally referenced as the main and most relevant mechanism involved in the release of DNA into extracellular space. However, this biased view is slowly being replaced by the understanding that there are several different pathways for the generation of cfDNA, many of which may contribute significantly towards the total pool of cfDNA. The major contributors include: (i) regulated, rapid or gradual cell death mediated by a variety of dedicated molecular mechanisms, such as necrosis, autophagy, and many other cell death subroutines, including NETosis, which results in the release of NETs, pyroptosis, necroptosis, ferroptosis, phagocytosis, mitotic catastrophe, anoikis, entosis, and parthanatos; (ii) accidental, rapid cell death caused by destructive chemical, physical, or mechanical perturbations; (iii) DNA-containing extracellular vesicles that can be released by both dying cells and live cells; (iv) active release of DNA from live cells [4,414,415,416,417,418,419,420,421,422], although evidence on the latter remains scant and is still under debate/investigation, and (v) release of mtDNA from cell-free respiratory competent mitochondria present in circulation [248].

As discussed earlier, the various cfDNA subtypes present in a typical biospecimen is genetically, epigenetically, and structurally diverse. While the above-mentioned cell death mechanisms are, for example, very well described in the literature, there are still very few studies on the correlation between the respective mechanisms of release from different contributing cells and their impact on the physico-chemical features of cfDNA. However, the limited research done to date suggests a strong correlation. It is generally reported that apoptosis generates mostly mono-nucleosomes with a smaller fraction of di- and tri-nucleosomes in some cases [423,424], while necrosis is reported to generate HMW cfDNA fragments (~10 Kbp) [425,426,427,428]. However, there is evidence that apoptosis can also result in the release of HMW cfDNA [429], while necrosis can result in the release of DNA populations that mirror apoptotic laddering and produce an abundant population of mono-nucleosomes [430]. The relative contribution of different mechanisms toward the pool of cf-mtDNA is not known; however, it is clear that cf-mtDNA fragments range in size between 40 and 300 bp [53,54,55,56]. This broad distribution is likely the result of enzymatic degradation coupled with a lack of protection by histone proteins, but it is not clear how much of this digestion takes place prior to release intracellularly or extracellularly after release. Concerning regulated cfDNA release, some reports suggest that DNA may be released from specific chromosomal regions during the division of genomically unstable cells. This DNA is postulated to be released by at least two different modes: (i) 1000–3000 bp fragments derived from micronuclei [378,379], and (ii) free-floating extrachromosomal circular DNA ranging between 30 and 20,000 bp [431,432,433,434]. Furthermore, as discussed earlier, there is also evidence of actively released EVs that are associated with DNA fragments ranging between 150 and 6000 bp [303,310,401].

### 9.3. Movement of cfDNA

The characteristics of cfDNA can be influenced by several conditions surrounding its movement from cells/tissues into body fluids. Taking cancer as an example, the level of cfDNA released by a tumor, as well as the rate at which cfDNA moves from the tumor into the circulatory system may depend on the unique histology, cellular turnover, vascularization, and perfusion of the tumor. These factors can also alter the pathway of cfDNA release. For example, hypoxia due to poor vascularization can result in increased rates of cell-death and the generation of more cfDNA [435]. Hypoxia may also shift the relative contributions of different modes of cell death, thereby altering the physico-chemical features of the cfDNA. Furthermore, the movement of cfDNA can also be influenced by biological barriers. For example, the blood-brain barrier may restrict the movement of cfDNA from brain tumors into the circulatory system. Indeed, higher levels of tumor-derived cfDNA has been found in cerebrospinal fluid (CSF) vs. plasma [79,80,117]. Similarly, a higher ratio of tumor-derived vs. background cfDNA may be found in body fluids that are in closer proximity to specific tumors, such as stool in colorectal cancers [436], cervical smear [437], or uterine lavage [438] in gynecological cancer, saliva in oral cancers [439], or urine in bladder cancers [440,441,442].

### 9.4. Extracellular Modifications, Stability, Clearance, and Half-Life

A variety of extracellular factors can affect the quantitative and qualitative properties of cfDNA. (i) While it is difficult to determine the physical properties of cfDNA at the instance of cellular release, several lines of evidence indicate that cfDNA fragments may be rapidly shortened by the action of different enzymes present in blood, such as DNase I [443,444] and DNase1L3 [445]. The most studied cfDNA size population in blood plasma is the mono-nucleosome with the most commonly reported size of 167–168 bp, representing 145–147 bp of DNA wrapped around the nucleosome core particle (NCP) plus linker DNA bound to histone H1 [415,417,446]. This size is likely due to Caspase activated DNase (CAD) digesting linker DNA 10 bp up- and downstream form the NCP [447]. The prevalence of the 167 bp mono-nucleosome in cfDNA studies suggests that the chromatosome is a stabilizing structure that protects DNA against enzymatic cleavage. However, CAD can also slide its digestion sites in increments of 5 bp in some cases [447], resulting in mono-nucleosome sizes of approximately 158, 163, 173, 178, 183, and 188, which are also observed in studies [430]. Among these, the 177–178 bp population is also often observed in studies [430,446], which is a size-population that has a higher probability of forming [448]. In addition to mono-nucleosomes, multiples of nucleosomal repeats are also often observed, e.g., di-nucleosomes, tri-nucleosomes, tetra-nucleosomes, and penta-nucleosomes. These cfDNA size populations also display modal sizes (i.e., 356 bp, 534 bp, 712 bp, and 890 bp, respectively), which seems to represent the size that is most likely to form under stochastic digestion of linker DNA [448]. The presence of these size populations is generally ascribed to apoptotic cleavage of chromatin. However, there is a growing number of studies reporting on the presence of HMW cfDNA present in body fluids [449,450,451,452,453,454,455,456,457] and cell cultures [305,414,415,416,417,430,458,459]. These HMW fragments may originate from apoptosis but may also be derived from many other release mechanisms, such as accidental cell lysis, necrosis, NETosis, regulated release through extracellular vesicles, and so forth. There is increasing evidence suggesting that these HMW cfDNA fragments may be digested into shorter fragments. While there may be some selectivity involved in cleavage locations, a generally stochastic inter-nucleosomal DNA digestion scheme seems likely. In such a scheme, the digestion of all longer fragments feeds all shorter fragments, which ultimately results in the rapid accumulation of mono-nucleosomal fragments. While more research is needed, this represents an alternative view to the view in which the presence of mono-nucleosomes is ascribed mainly to apoptosis. In addition to HMW cfDNA and mono-nucleosomes, a growing number of studies are demonstrating the presence of sub-nucleosomal and ultrashort, single-stranded cfDNA fragments in body fluids [106,184,252,460,461,462,463,464]. Whether these shortened cfDNA fragments represent short fragments released from cells or extracellularly digested products of longer fragments is not yet clear. However, given the presence of proteolytic enzymes in blood, it is likely that a large proportion of these short fragments represent DNA derived from destructed nucleosomes. (ii) In addition to enzymatic or proteolytic degradation, cfDNA fragments can either be captured and digested by the liver and spleen [465,466], or be absorbed by the kidneys followed by urinary elimination [467,468,469]; (iii) CfDNA molecules can also attach to the membranes of circulating cells (e.g., red and white blood cells) or endothelial cells, where they may either (a) remain attached for prolonged periods or be digested by enzymes, (b) be assimilated into the cells, or (c) attach and detach dynamically depending on pH, temperature, and the influence of various substances (reviewed in ref. [470]); (iv) the enzymatic/proteolytic degradation, uptake by organs for digestion or excretion, and cellular internalization of cfDNA may depend significantly on its physico-chemical features, many of which may be altered by the binding of cfDNA molecules to EVs, circulating DNA-binding proteins (e.g., high density lipoprotein, argonaute 2, albumin, fibrin, fibrinogen, prothrombin, transferrin, globulins, C-reactive protein, and serum amyloid A), and other macromolecules. It is important to note here that (1) most of the factors discussed above have not yet been studied extensively, (2) the relative contribution of each factor towards the stability and clearance of cfDNA is not known, (3) we still have a poor understanding of the processes involved in the generation of cfDNA, and (4) many of these factors are non-mutually exclusive. This makes it very difficult to establish the dynamics and baseline characteristics of cfDNA. For example, while the half-life of cfDNA is estimated between 16 min and 2.5 h [75,76,77], there is no actual consensus, as this half-life refers to the stability of mono-nucleosomal cfDNA and not the total cfDNA population.

### 9.5. Summary

As discussed earlier, a wide range of disorders, physiological states and factors, and clinical scenarios result in increased cfDNA levels. Among these, numerous factors (e.g., mechanical stress, oxidative stress, hypoxia, inflammation, DNA damage, genomic instability, lesions, etc.) may trigger different types of cellular demise or alter constitutive DNA release. Similarly, many cfDNA-modifying factors such as the extracellular concentration of enzymes and DNA-binding proteins, or the rate of cfDNA uptake by the liver, spleen, and kidneys are augmentable by various factors (e.g., time of day, body mass index, diet, medication, fitness, exercise, metabolic rate, etc.) many of which are liable to significant intra- and interindividual variation. As many of these factors may not only occur simultaneously but are also inextricably linked in a specific pathological or physiological state, it has to-date been virtually impossible to make concrete correlations between a specific factor and the dynamics or characteristics of one cfDNA subtype.

## 10. Characterization of cfDNA in Animals

Beyond the role of cfDNA in humans, cfDNA has been investigated in various animals with an overrepresentation of studies on dogs. While cfDNA research on non-human animals is not as developed as in humans, there is increasing interest in the role of cfDNA as a biomarker as well as a tool to study pathophysiology in animals, especially companion animals. For example, several studies indicate the potential of implementing cfDNA tests in veterinary clinics for the minimally-invasive diagnosis and monitoring of cancer in dogs [471,472,473,474,475,476,477]. Furthermore, cfDNA may also serve as a potential marker of gastric dilatation–volvulus (GDV) [478] and immune-mediated hemolytic anemia (IMHA) [479], tissue injury [480], and sepsis [481,482,483] in dogs. Additionally, similar to humans, strenuous exercise has been shown to correlate with elevated levels of cfDNA in dogs [484]. Besides dogs, one study has measured cfDNA in cats with diffuse iris melanomas and concluded that cfDNA levels and integrity are not sufficient markers for diagnosis and prognosis [485]. Interestingly, the characterization of cfDNA derived from the microbiome of non-human primates may allow the discovery and monitoring of taxa that cause zoonotic diseases [108,486]. Furthermore, various animal models have been used to study correlations between different pathological factors and corresponding changes in cfDNA, which may have implications in human biology and pathology, such as the study of traumatic brain injury in rats [487], carbon tetrachloride-induced acute liver injury in rats [488], perinatal asphyxia in piglets [489], tumor progression and metastases in rats [490,491], the onset of parturition in mice [492], and gateways regulating the release of tumor-derived cfDNA in mice [493].

## 11. Perspectives on the Importance of an Improved Understanding of cfDNA Biology

**Figure 8:**
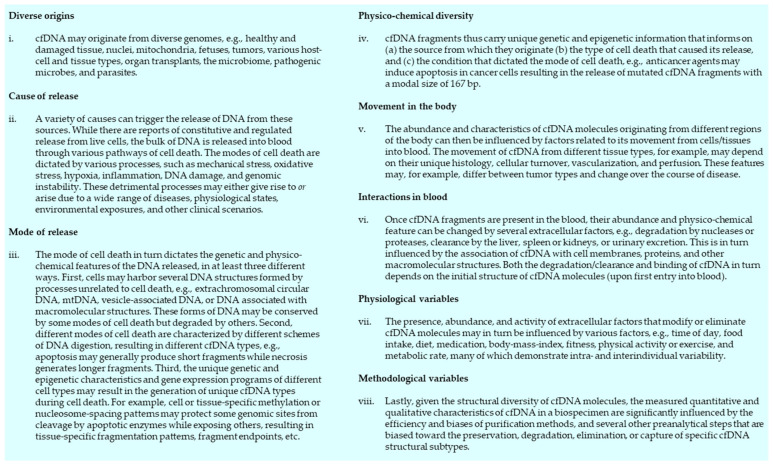
Factors that contribute to the complexity and fluctuation of cfDNA composition.

In this review, we showed how the composition of cfDNA in blood is both highly complex and changeable. Several overlapping phenomena contribute to this (Figure 8).

From the points outlined in Figure 8, a picture of immense complexity emerges. The factors that modulate the observed properties of cfDNA are vast, inextricably linked, and are riddled with overlapping characteristics and circular causation. Not only do we currently have a weak grasp on the outlines of this problem, but also a very poor understanding of most of the individual factors. Yet, it is increasingly recognized that a holistic understanding of cfDNA biology is vital [1,3,6,59,130,137,253,494]. A deep and structured enquiry into the origin, physico-chemical features, and functions of cfDNA as it relates to the various biological, physiological, pathological, environmental, and lifestyle factors as well as preanalytical factors will provide new insights that impact many aspects of cfDNA research.

### 11.1. Increasing the Sensitivity and Specificity of Existing cfDNA Assays

In a highly heterogeneous cfDNA population, both the absolute amount and relative proportions of most target molecules are often low. However, elucidation of the various factors that respectively affect the concentration of target molecules and background cfDNA molecules in a biospecimen will on the one hand inform the selection of optimal preanalytical procedures and patient conditions, and on the other hand facilitate the development of methodologies that either maximizes the absolute number of target molecules or increases the ratio of target-to-background cfDNA molecules, thereby significantly increasing the chances of detection.

For example, the amount of target molecules in a sample may be increased by (a) selecting the right biospecimen type (e.g., target molecules may be enriched in body fluids that are in closer proximity to the source of the target molecules of interest, such as ctDNA in the CSF of patients with brain tumors); (b) selecting patient conditions that (i) favor the release of target molecules, (ii) limit the release of background molecules, or more likely (iii) enhance the release of total cfDNA. In line with this, it can be conjectured that an improved understanding of the mechanisms involved in the release of cfDNA may permit the use of compounds to enhance or limit cfDNA release. However, this approach may be offset by the potential detrimental effects of dramatically shifting cfDNA levels (as discussed in Section 8); (c) the use of tailored extraction procedures developed for the capture of specific target molecules or elimination of non-target molecules, as informed by knowledge of the exact structural features or chemical properties of specific cfDNA molecules. The efficacy of cfDNA biology-informed methods is demonstrated by (1) a study that has achieved greater cancer assay sensitivity and specificity through experimental capture or in silico selection of mutant-enriched short cfDNA fragments [184]; (2) a study that demonstrated the possibility of selectively capturing mutant molecules based on cancer-specific DNA methylation aberrations with enhanced affinity for gold nanoparticles [495]; (3) studies that have shown that exosomes often carry cargo enriched in tumor material. Besides the aforementioned examples, cfDNA molecules come in a variety of shapes and sizes, many of which are affected in different ways by various preanalytical steps. An exploration of these differences is not only likely to reveal the ideal preanalytical workflows for specific cfDNA molecules, but will also inform the development of new and more effective products and methods.

### 11.2. Expanding the Repertoire of Disease-Specific cfDNA Markers

As outlined in Section 5, a growing body of clinical studies shows strong correlations between epigenetic features of cfDNA and disease dynamics in various pathologies, especially cancer. By virtue of the increased proportionality of epigenetic modifications vs. single-gene mutations, epigenetic cfDNA assays often show increased diagnostic sensitivity and specificity, especially for the characterization of early-stage disease. While there is currently limited evidence of its clinical validity, and many preanalytical obstacles to overcome, there is little doubt among researchers and clinicians that cfDNA testing will be integrated into standard patient care in the near-future. While the majority of cfDNA applications in oncology are still centered on the detection of single-gene mutations in cfDNA, the focus of cfDNA research is slowly shifting towards epigenetic characterization. Indeed, the major efforts that are currently being made to characterize tissue- and disease-specific epigenetic markers of cfDNA will lead to the development of new and more powerful assays that significantly expand the liquid biopsy toolbox. This represents an entirely new modality in the application of cfDNA assays and will play a significant part in the transformation of personal molecular medicine through cfDNA profiling.

### 11.3. Enable the Characterization and Study of Temporal Genome Dynamics

An expanded knowledge of cfDNA biology will enable increasingly high-fidelity reconstruction of the physiological, pathological, and cell- and tissue-specific information harbored by cfDNA molecules. Robust longitudinal profiling of cfDNA may allow minimally invasive characterization of temporal genomic changes in specific regions of interest in response to a wide variety of factors. In contrast to traditional methods of genome analysis (e.g., cell cultures, in vivo models, and tissue biopsies), which have been valuable but limited by the inherent restraints of reductive approaches (e.g., sampling bias, loss of contextual logic, snapshot information) (reviewed in ref. [130]), data from serially profiled cfDNA represent a richer, more dynamic and much more realistic source of actual genome function, composition, and alterations.

Therefore, the genomic information accessible through serial cfDNA analysis can be mined for (i) the discovery of new and increasingly powerful combinations of surrogate markers for various disease indications, and (ii) unprecedented analysis of genome function and systematic mapping of both benign and malignant genetic and epigenetic alterations caused by a variety of biological, physiological, pathological, and environmental factors. Research on temporal genomic characterization through cfDNA is still in its infancy, with many challenges ahead. However, the successes achieved in temporal analyses of tumor genomes have moved theoretical deliberations from the realm of fantasy and speculation into concrete evidence and practical utility. For example, serial cfDNA assays have been used to detect and monitor minimum residual disease, metastases, intratumor genetic heterogeneity, shifting mutational landscapes, genetic responses to chemo- and radiotherapy, and mechanisms that underlie the emergence of therapy resistance. There is strong evidence that this broad information, which is virtually unattainable through tissue biopsies, can be leveraged to improve diagnoses, prognoses, guide and improve therapeutic regimes, limit overtreatment and risk of recurrence, and ultimately improve patient quality-of-life and overall survival. Another example of the promising results achieved by serial analyses of cfDNA is the monitoring of donor-derived cfDNA in organ transplant patients to detect signs of rejection or injury of the transplanted organ. While much clinical validation is needed before these cfDNA assays will be widely implemented, the progress made in the field of oncology and organ transplant monitoring indicates the possibility of developing cfDNA assays for monitoring a potentially wide range of pathologies and other clinical scenarios.

Serial cfDNA analyses may have many other potential uses. (i) They may provide new mechanistic insights into the pathological events underpinning many slowly progressive or chronic illnesses, especially those that have been correlated with cell death and aberrant cfDNA profiles, such as CVDs, diabetes, autoimmunity, and neurodegenerative disease; (ii) they may be especially useful for monitoring progressive diseases or clinical scenarios that cause rapid genomic alterations at specific time-points, such as Parkinson’s disease, Alzheimer’s disease, sepsis, stroke, traumatic injuries, and malfunctioning gene therapy; (iii) they may be used to monitor the safety and efficacy of various kinds of therapy; (iv) they may be useful for studying the diversity and fluctuation of the microbiome as well as its role in human health and disease; (v) they can be used to trace the biological footprint and effects of assimilated environmental DNA; (vi) they can be used to study complex biological phenomena such as genomic mosaicism; (vii) they can be used to investigate the dynamic response of the genome in general or the genome of specific organs to the diet (e.g., long-term consumption of inflammatory foods) or dramatic dietary changes; and (viii) they can be used to determine body-wide changes of the genome as a result of aging. In addition to structural genomic changes, an increasing number of studies indicate that cfDNA can be used to infer gene expression patterns in specific tissues. Refinement of such approaches in concurrence with an improved understanding of cfDNA biology may, in the future, allow the use of cfDNA for studying benign and detrimental gene expression in various contexts, e.g., disease, exercise, and poorly understood biological processes (e.g., embryogenesis).

While the use of cfDNA for these purposes is mostly speculative at this stage and depends highly on the rate at which we expand our knowledge of cfDNA biology, its potential to transform our understanding of genome biology and pathology is undeniable and should, therefore, be provoking more comment than it currently is.

## 12. Concluding Remarks

**Figure 9:**
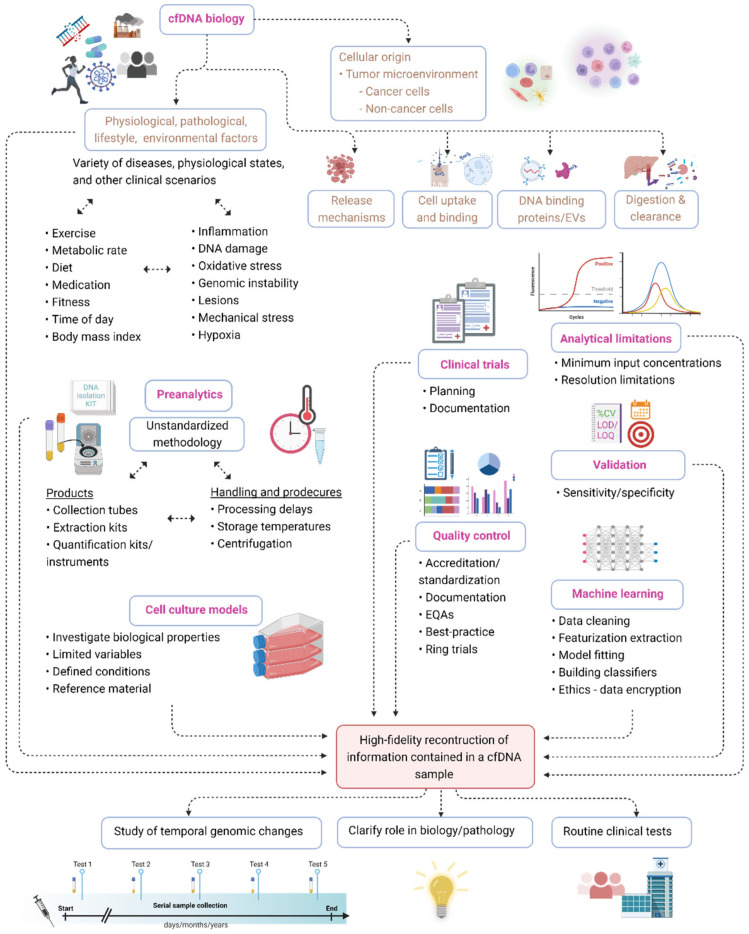
Factors that need to be addressed in order to increase the fidelity of cfDNA analysis. In order to (i) use cfDNA for studying temporal genomic changes, (ii) investigate the role of cfDNA in human health and disease, and (iii) develop increasingly powerful clinical cfDNA assays, the quantitative and qualitative information contained in cfDNA samples needs to be reconstructed with high fidelity. Here we briefly summarize various factors that need to be considered and steps that can be taken towards increasingly accurate cfDNA measurements.

While considered trivial at the time of its discovery and even decades thereafter, cfDNA is now emerging as one of the most interesting classes of molecules in human biology. In this review we showed how cfDNA is uniquely poised to transform clinical diagnostics as well as genomics research in various ways: (i) it may serve as highly specific and versatile biomarkers for a wide range of pathologies; (ii) it may serve as surrogate markers for monitoring or studying numerous physiological states; (iii) it may grant an unprecedented window of access to characterize temporal genomic alterations in numerous contexts; and lastly (iv) as cfDNA molecules have biological activity and may contribute to both pathology and normal biological functions, it may possibly shed new light on some of the unknown or poorly understood mechanisms underlying various pathologies and important biological processes. However, several challenges need to be overcome if we want to harness the full potential of cfDNA for these various purposes (Figure 9).

Among the potential applications of cfDNA, its perusal as a biomarker has received the most attention. However, despite the spectacular results that have been demonstrated by clinical studies in various domains of medical research, only a small number of cfDNA assays have to date been validated for clinical use, and only in the fields of oncology and prenatal testing. The factors that challenge the development and clinical implementation of cfDNA assays are multifarious and overlapping:

As it is a neglected factor in cfDNA research, we, in this review, shed some light on how the immense heterogeneity and fluctuation of the cfDNA population in vivo, and by extension in biospecimens, is the unique factor that concomitantly makes cfDNA such a valuable biomarker class and tool in basic biological and genomic research, but also a very challenging analyte. High-fidelity reconstruction of the information encoded into the aggregate cfDNA population, or specific cfDNA target molecules, requires a significantly improved understanding of the genetic and physico-chemical features of cfDNA as it relates to various biological, physiological, pathological, lifestyle, environmental, and preanalytical factors. This may prove to be very challenging, but useful insights may also be achieved using in vitro methods. For example, in studying the biological factors that affect the properties of cfDNA, many of the inherent complexities of the in vivo milieu may be circumvented by using cell culture models. While much context about the in vivo setting may be lost in cell culture investigations, significantly diminished variables may allow unique insights into the biological properties of cfDNA in vivo. Another approach may be to monitor the physico-chemical properties and fluctuation of a purified cfDNA subtype spiked into plasma biospecimens or into artificial/simulated plasma.

Nevertheless, the heterogeneity and fluctuation of cfDNA is only part of the problem. While an in-depth enquiry into these factors were beyond the scope of this review, it is worth noting that the developmental timeline for clinically meaningful tests is extended by several persistent obstacles that need to be overcome, including a lack of universal preanalytical standards, an ever-expanding menu of preanalytical products, limited best practice guidelines, analytical limitations, no standard reference materials, insufficient analytical validation, and poorly-designed clinical trials. In addressing each of these obstacles, it would be beneficial to develop strategies for assessing the influence of the biological factors of cfDNA.

Moreover, it is becoming increasingly clear that new bioinformatics approaches, such as the incorporation of ML algorithms, are desperately needed to interpret and understand the sheer amount of data generated in cfDNA research, especially studies in which numerous parameters are assessed.

## Figures and Tables

**Table 1:** Developments that demonstrate the potential clinical utility of sequence-specific cfDNA assays.

Development	Use/Implementation
Food and Drug Administration (FDA)-approved cfDNA assays for use in routine diagnostic settings	BRCA1 and BRCA2 mutations in metastatic castration-resistant prostate cancer
	EGFR mutations in non-small cell lung cancer
	KRAS G12C mutations in non-small cell lung cancer
	PIK3CA mutations in breast cancer
CfDNA Assays performed in Clinical Laboratory Improvement Amendments (CLIA)-certified laboratories	CtDNA assays that have been validated in CLIA-approved clinical laboratories are increasingly offered to cancer patients worldwide
CfDNA assays performed in non-invasive prenatal testing (NIPT) facilities	NIPT facilities worldwide offer cfDNA-based tests for the screening and early characterization of various fetal characteristics, such as sex and chromosome conditions, i.e., aneuploidy, trisomies, and microdeletions

**Table 2:** Classifications of different types of extracellular vesicles.

Name	EV Class	Size (nm)	Biogenesis	DNA Content	DNA Localization
Exosome	small EV	40–100	Multivesicular endosome	DNA, mtDNA, viral	outer surface
Microvesicle	large EV	~100–1000	Plasma membrane budding	dsDNA	lumen
Large oncosome	large EV	1000–10,000	Plasma membrane budding	ssDNA/dsDNA	unknown
ARMM	small EV	~40–100	Plasma membrane budding	None found	N/A
Apoptotic body	large EV	500–2000	Apoptosis	dsDNA/mtDNA	lumen
Exomere	non-EV	~35–50	Unknown	DNA detected	unknown
Micronucleus	large non-EV	1000–9000	Mitotic catastrophe	gDNA	lumen
OMV	small EV	20–250	Outer membrane budding	dsDNA	lumen and surface

**Table 3:** Cell-free DNA (cfDNA) studies that employed machine learning.

CfDNA Feature Characterized	Machine Learning Algorithm Applied	Reference
Aneuploidy, amplicons	SVM, LR	[356]
Concentration of cfDNA, fragmentation, methylation	JADBio, SVM, LR, RF (bagged tree)	[353]
Coverage	LR	[358]
CNVs and fragmentation patterns	SVM, LR	[346]
CNVs and fragmentation patterns	SVM	[180]
CtDNA mutations	Hierarchical Bayesian noise model score, spearman’s correlation	[355]
CtDNA mutations (loci)	RF	[359]
CtDNA mutations and CNV	Ensemble of 5nn, 3nn, Naive Bayes, LR, Decision Tree	[357]
CtDNA mutations and proteomics	LR	[332]
Fragment ends	RF	[360]
Fragment sizes, coverage	Gradient Tree Boosting	[185]
Fragment sizes, t-MAD, fragment size distribution, fragment profile amplitudes	LR, RF	[184]
Methylation	Clustering UPGMA, dynamic programming	[175]
Methylation	RF	[354]
Methylation	RF	[351]
Methylation	RF, Gaussian model-based Mclust	[154]
Methylation	SVM	[348]
Methylation and fragmentation	SVM	[350]
Methylation, miRNA	RF	[347]
Methylation, top 300 dmr windows of 300 bp	RF	[158]
Somatic point mutations, copy number alterations	SVM. LR, RF	[349]

Abbreviations: SVM: support vector machine; LR: Logistic Regression; RF: Random Forest.
